# Engineering new balancer chromosomes in *C. elegans* via CRISPR/Cas9

**DOI:** 10.1038/srep33840

**Published:** 2016-09-21

**Authors:** Satoru Iwata, Sawako Yoshina, Yuji Suehiro, Sayaka Hori, Shohei Mitani

**Affiliations:** 1 Department of Physiology, Tokyo Women’s Medical University School of Medicine, Tokyo, Japan; 2 Tokyo Women’s Medical University Institute for Integrated Medical Sciences, Tokyo, Japan

## Abstract

Balancer chromosomes are convenient tools used to maintain lethal mutations in heterozygotes. We established a method for engineering new balancers in *C. elegans* by using the CRISPR/Cas9 system in a non-homologous end-joining mutant. Our studies will make it easier for researchers to maintain lethal mutations and should provide a path for the development of a system that generates rearrangements at specific sites of interest to model and analyse the mechanisms of action of genes.

Genetic balancers (including inversions, translocations and crossover-suppressors) are essential tools to maintain lethal or sterile mutations in heterozygotes. Recombination is suppressed within these chromosomal rearrangements. However, despite efforts to isolate genetic balancers since 1978^1^,^2^,^3^,^4^,^5^, approximately 15% (map units) of the *C. elegans* genome has not been covered^6^ (Supplementary Fig. 1). Because the chromosomal rearrangements generated by gamma-ray and X-ray mutagenesis are random, it is difficult to modify specific chromosomal regions. Here, we used the CRISPR/Cas9 genome editing system to solve this problem. The CRISPR/Cas9 system has enabled genomic engineering of specific DNA sequences and has been successfully applied to the generation of gene knock-outs and knock-ins in humans, rats, mice, zebrafish, flies and nematodes^7^. Recently, the CRISPR/Cas9 system has been shown to induce inversions and translocations in human cell lines and mouse somatic cells^8^,^9^,^10^. Similarly, inversions up to 57.5 kb have been obtained in the zebrafish germline^11^. Although a large number of cells can be treated at once for effective CRISPR/Cas9 editing in cell lines, it is more difficult to do so in the germlines of model organisms because of limitations in the ability to introduce genome editing tools. Thus, researchers need an efficient way to engineer the chromosomal structure in multicellular organisms *in vivo*. In the present study, we established an editing method using the CRISPR/Cas9 system in *C. elegans* to generate genetic balancers at specific chromosomal sites. The inversions and crossover-suppressors produced were up to 6.7 Mb (~17 cM), lengths 2 orders of magnitude longer than produced in a previous work in the germline of a model organism. To facilitate genomic engineering, we targeted the genome rearrangements in a non-homologous end-joining (NHEJ) mutant background. Our method resulted in a higher proportion of successful rearrangements to generate new balancers. Moreover, we found that the inversion and crossover-suppressor balancers generated in heterozygotes did not result in interchromosomal effects.

## Results and Discussion

### Experimental design to screen for new balancers on chromosome IV

**Figure 1:**
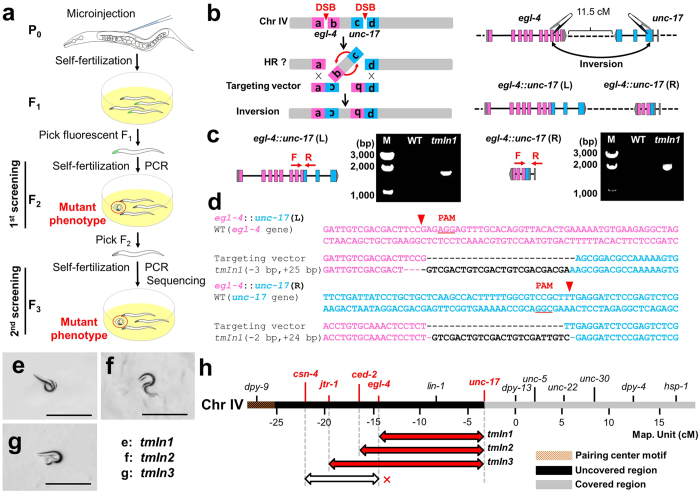
Genetic engineering of new balancers by using the CRISPR/Cas9 system. (**a**) Experimental design to screen for inversion balancers. (**b**) Schematic of the chromosomal rearrangement *tmIn1. tmIn1* was created by an inversion between *egl-4* and *unc-17*. (**c**) PCR amplification of breakpoint junctions in wild-type (WT) and *tmIn1* animals. (**d**) Breakpoint sequence alignments of the targeting vectors and *tmIn1* rearrangement. Black bars indicate the cleavage sites. (**e**) The relative positions of breakpoints on chromosomal balancer IV. The generated balancers are indicated by red double-headed arrows. A white arrow with a cross indicates a failed trial. (**f**,**g**,**h**) Generated balancers showed a recessive larval arrest phenotype. Scale bars represent 100 μm.

We designed two sgRNAs (single guide RNA) in the exons of two target genes that result in easily identifiable phenotypes when they are disrupted. We next constructed two targeting vectors that joined the chromosomal breakpoints together, each of which had 2 kb of sequence homologous to each predicted junction point, so that chromosomal rearrangements could be induced by homologous recombination (HR) between the targeted regions and homology vectors (Fig. 1b, Supplementary Fig. 2). A previous study has reported that disabling NHEJ via the RNAi inactivation of the *cku-80* gene (a homologue of the human *KU80*), which acts as a DNA binding protein, significantly improves the HR efficiency in *C. elegans*^12^. Therefore, NHEJ disruption may allow for the efficient repair of DSBs by using targeting vectors via HR. One of the NHEJ genes, *lig-4* (a human *LIG4* homologue), is essential for the final ligation step of the DNA ends. A strain known to contain a disruption in *lig-4*, *tm750*, was used in the experimental procedures and is depicted in Fig. 1a.

We first attempted to generate an inversion balancer on the left arm of chromosome IV (Fig. 1b), which includes part of the largest region of the *C. elegans* genome that is not covered by current genetic balancers (Supplementary Fig. 1). We co-injected sgRNAs targeting *egl-4* and *unc-17* with Cas9, the targeting vectors and a *Pmyo-2::Venus* transgene marker into the gonads of young adult P_0_ worms. We screened for fluorescent F_1_ worms that contained larval-arrested F_2_ progeny caused by *unc-17* disruption and confirmed the rearrangements by PCR amplification of both junction points. To examine whether the candidates maintained the chromosomal inversion, F_2_ worms that laid larval-arrested F_3_ progeny were further investigated by using both PCR amplification and DNA sequencing. Through these experimental procedures, one chromosomal rearrangement was obtained in each of the 136 F_1_ worms in the *lig-4*(*tm750*) mutant background (Table 1). The rearrangement named *tmIn1*(*IV*) exhibited detectable *egl-4::unc-17* fused genes at two junction points, as confirmed by PCR amplification of the breakpoints, but the WT did not (Fig. 1c). These breakpoints were verified by DNA sequencing (Fig. 1d). The rearrangement *tmIn1*(*IV*) exhibited a recessive larval arrest phenotype (Fig. 1e). Thus, these experimental techniques induced successful chromosomal rearrangements in the germline of a multicellular organism.

We further screened for the generation of large chromosomal balancers on the left arm of chromosome IV. We obtained two additional balancers, *tmIn2*(*IV*) and *tmIn3*(*IV*), which covered 13.5 cM and 16.4 cM, respectively (Fig. 1h, Table 1, Supplementary Figs 3 and 4). The probability of obtaining inversion strains appeared to decrease as the target size became larger (0.60% and 0.32% for *tmIn2* and *tmIn3*, respectively: Table 1). The *tmIn2*(*IV*) and *tmIn3*(*IV*) worms exhibited a recessive larval arrest phenotype (Fig. 1f,g). Although target sites between *csn-4* and *egl-4* covering only 7.6 cM and sgRNA-specific mutations were observed, these chromosomal rearrangements could not be isolated (Fig. 1h, Table 1). One of the target genes (*csn-4*) was located near pairing centres (PCs), where the chromosome is stabilized by homologue pairing^13^. The generation frequency decreased at the end of the PC side of the chromosome (Table 1). Heterochromatin is important for maintaining the structural integrity of the genome^14^. However, *tmIn1*(*IV*), *tmIn2*(*IV*) and *tmIn3*(*IV*) rearrangements were generated on regions of the chromosome arm that are known to contain heterochromatin^15^ (Table 1). These results suggest that our approach can generate chromosomal rearrangements at specific sites even in heterochromatic regions, whereas these rearrangements were below the generation limit mainly because of the presence of PCs.

### Confirmation of the suppression of recombination in *tmIn3*(*IV*)

We examined whether *tmIn3*(*IV*) could balance a recessive lethal mutation within the inversion interval, as described in Supplementary Fig. 5a. Heterozygous *tmIn3*(*IV*) hermaphrodites were mated with heterozygous males carrying a recessive lethal *lin-1* mutation (*tm5929*). After the self-fertilization of F_1_ worms, the balanced strain *lin-1/tmIn3*(*IV*) segregated three phenotypes: WT (*lin-1/tmIn3* heterozygotes), lethal (*lin-1* homozygotes) and larval arrest (*tmIn3* homozygotes) (Supplementary Fig. 5b–g). Thus, the new balancer is a useful tool for maintaining lethal mutations on the left arm of chromosome IV. The segregation of these phenotypes was maintained through more than 20 generations, suggesting that *tmIn3* suppresses further recombination of the covered genomic region.

### Whole-genome sequence analysis of *tmIn3*(*IV*)

To further assess whether the generated balancer strains exhibited rearrangements at unexpected loci, we extracted the genomic DNA from the *lin-1*(*tm5929*)*/tmIn3*(*IV*) worms and analysed it by whole-genome sequencing (Supplementary Fig. 6, Supplementary Table 1). We observed several structural variants consistent with target-specific rearrangements but no target-independent rearrangements. From these results, we conclude that our methodology can accurately induce target-specific rearrangements.

### Isolation of an inversion balancer on chromosome II

Likewise, we sought to generate a chromosomal rearrangement on the left arm of chromosome II, which includes the second-largest region of the *C. elegans* genome that is not covered by known balancers (Supplementary Fig. 1). We obtained a new balancer named *tmIn4*(*II*), which covered 8.6 cM and extensively maintained recessive lethal mutations (Supplementary Figs 7 and 8, Supplementary Table 2). Thus far, we have not been able to generate a balancer near the PCs on chromosome II (Supplementary Fig. 7e, Supplementary Table 2). The results from chromosome IV also indicate that successful chromosomal rearrangements are mainly affected by the distance from the PC region (Fig. 1h).

### Genetic engineering of crossover-suppressor and translocation balancers

**Figure 2:**
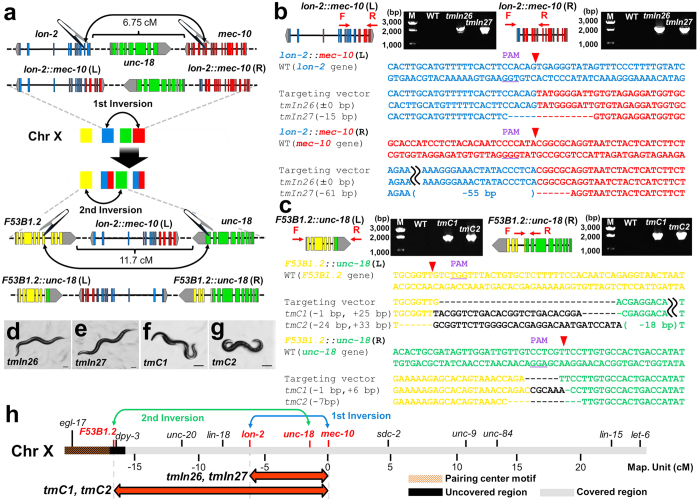
Genetic engineering of crossover-suppressors by using the CRISPR/Cas9 system. (**a**) Schematic of a crossover-suppressor. The Crossover-suppressor was created by the multiple inversions. (**b**) PCR amplification of the breakpoint junctions in wild-type (WT), *tmIn26* and *tmIn27* animals. Breakpoint sequence alignments of the targeting vectors and *tmIn26* and *tmIn27* rearrangements. Black bars indicate the predicted cleavage sites. (**c**) PCR detection of the breakpoint junctions in WT, *tmC1* and *tmC2* animals. Breakpoint sequence alignments of targeting vectors and *tmC1* and *tmC2* rearrangements. (**d**) The relative positions of breakpoints in the X chromosomal balancers. (**e**,**f**) *tmIn26* and *tmIn27* showed a recessive long phenotype. (**g**,**h**) *tmC1* and *tmC2* showed a recessive uncoordinated phenotype. Scale bars represent 100 μm.

In addition to the generation of inversions, we also engineered two crossover-suppressors, *tmC1*(*X*) and *tmC2*(*X*), composed of sequential inversions between *lon-2* and *mec-10*, and between *F53B1.2* and *unc-18* (Fig. 2, Supplementary Table 3). This strategy was used to produce more stable balancers because multiple inversions more effectively prevent recombination^6^. The crossover suppressors *tmC1*(*X*) and *tmC2*(*X*) covered most of the left arm of the X chromosome from *F53B1.2* to *mec-10*, covering 17 cM (Fig. 2h).

Next, to examine whether our method was also effective between different chromosomes, we generated a chromosomal translocation named *tmT3*(*III;IV*) that arose between *pal-1*(*III*) and *unc-17*(*IV*) (Supplementary Fig. 9). There were no differences in the efficiency of generating inversions or translocations (Table 1, Supplementary Fig. 9d; Fisher’s test, P > 0.05). Thus, the results demonstrate that these experimental methodologies successfully provided a systematic approach to target chromosomal rearrangements at specific sites. Because the DNA repair pathways are highly conserved across species, our methodology may provide a universal approach for engineering chromosomal rearrangements.

### Generation of transgene-integrated strains by using the CRISPR/Cas9 system

To facilitate balancer chromosome usage, we developed a technique using the CRISPR/Cas9 system that produced multi-copy fluorescent gene integration in *tmC1*(*X*) from extrachromosomal arrays^16^ (Supplementary Fig. 10a). We first generated an extrachromosomal line *tmC1;tmEx4487* that expressed *Pmyo-2::Venus* together with *Punc-18::unc-18* (which rescues *unc-18* disruption) and a *dpy-3* genome sequence as the sgRNA target. We co-injected the *dpy-3* genome sequence-specific sgRNA with a Cas9 expression vector and a *Pdpy-7::DsRed* transgene marker into the gonads of *tmC1;tmEx4487* worms. We isolated F_1_ worms with Venus and DsRed expression and screened F_2_ progeny for dumpy (Dpy) phenotypes and Venus expression. The breakpoints were examined by PCR amplification (Supplementary Fig. 10b). Venus fluorescent Dpy worms that carried the balancer chromosome and harboured the *Pmyo-2::Venus* transgene were isolated as *tmC1*[*F53B1.2 lon-2 unc-18 mec-10 Pmyo-2::Venus Punc-18::unc-18*] (Supplementary Fig. 10c,d).

### Examination of the repair mechanisms that generate rearrangements

During the course of isolation of genetic balancers, we found that only a portion of the phenotype-positive lines yielded PCR-positive alleles; in the case of *tmIn1*, only 6 of the 40 phenotype-positive lines were PCR-positive (Table 1). This finding implies that DSBs are often repaired without inversion or with structural changes that are unable to be amplified by PCR, thus suggesting that breakpoints are often repaired by a mechanism other than HR. To determine whether the targeting vectors and *lig-4* mutant background were truly necessary, we injected the genome-editing and marker plasmids without targeting vectors in the gonads of WT and *lig-4* worms. In the absence of the targeting vectors, we still obtained inversions (in 0.33% of WT and 0.13% of *lig-4*(*tm750*) offspring), but the probability was decreased compared with that observed in the *lig-4*(*tm750*) background injected with targeting vectors (0.73%). Recent studies have shown that the CRISPR/Cas9-induced DSB repair of germ cells in *C. elegans* is often mediated by polymerase theta-mediated end-joining (TMEJ)^17^. These observations suggest that in the *lig-4*(*tm750*) mutant background, the targeting vectors may be required for HR, but TMEJ may also induce rearrangements. A previous report has also identified the generation of inversions that depended on the *LIG4* gene in human cells without repair templates^18^, suggesting that NHEJ may also be involved in the process.

Upon closer inspection of the repaired regions in the rearrangements in 9 strains obtained from the *lig-4* mutant background by using targeting vectors, we found that only one strain (*tmIn26*) contained complete copies of the targeting vector sequences at both breakpoints (Supplementary Table 4). Another strain *tmT3* contained one complete copy at a breakpoint but contained an indel at another breakpoint. The other 7 strains had indels at both breakpoints. Thus, of 18 breakpoints, 3 appeared to be repaired by HR, whereas 15 were repaired by TMEJ. This phenomenon suggests that each breakpoint is repaired by either system stochastically.

In the case of 4 inversion strains without targeting vectors, we found that all the breakpoints contained some indels of the genome sequence (Supplementary Table 4 and Supplementary Fig. 11). This result suggests that TMEJ (for these 4 strains) or NHEJ (except for the case of *tmIn45*), might be used to repair the breakpoints.

It should also be noted that we were unable to obtain any inversions in the wild-type background by using targeting vectors (0/755 F_1_ animals). Although it is expected that all three repair mechanisms (HR using targeting vectors, NHEJ and TMEJ) can repair breakpoints, we could not find any evidence for successful rearrangements among the 102 phenotype-positive candidates (Table 1). The probability of successful rearrangements appeared even lower than that in the wild-type background without targeting vectors. Although the mechanisms for this phenomenon remain unclear, we speculate that the introduction of targeting vectors could mobilize NHEJ, thus quickly resulting in the repair of breakpoints without inversion^19^,^20^.

## Conclusion

In summary, our strategy systematically generated chromosomal inversion, translocation and crossover-suppressor balancers at specific sites. These new balancers covered 8% of the *C. elegans* genome, remaining 7% of the 15% of the genome that was previously uncovered by balancers. It should be noted that our crossover-suppressor lines containing a fluorescent marker are ideal for the analysis of lethal mutations. Many of the common balancer lines used by the *C. elegans* research community have translocations and thus suffer from aneuploidy, which is inconvenient for phenotypic analyses^6^. In contrast, inversion and crossover-suppressor balancer lines have structural variations within their own chromosomes, are free from aneuploidy and are more straightforward to use for the examination of mutant phenotypes. Unfortunately, the crossover-suppressors used to date in the field have complex chromosomal structural changes. Our strategy using CRISPR/Cas9 resulted in minimal additional chromosomal changes. Our crossover-suppressors with double inversions covered a larger genomic region than did simple inversion balancers. Finally, we were able to introduce locus-specific fluorescent markers into these crossover-suppressor lines^16^.

## Methods

### Nematode strains

*Caenorhabditis elegans* wild-type strain Bristol N2 was used in this study. Lines carrying *lig-4*(*tm750*), *lin-1*(*tm5929*), and *mlt-7*(*tm1794*) mutations were obtained previously^21^. Nematodes were grown by using standard genetic protocols^22^.

### Plasmid construction

We used site-directed mutagenesis to insert the guide sequences into a Peft-3::Cas9 + sgRNA dual expression vector (pDD162, Addgene plasmid 47549, Cambridge, MA). We designed G(N)_19–25_NGG specific sgRNA sequences as previously described^23^ (Supplementary Table 5). The sgRNA sequences were designed to target the exons of genes with easily identifiable loss-of-function phenotypes, such as uncoordinated (Unc), dumpy (Dpy), long (Lon), or lethal (Let). The Cas9-sgRNA plasmids were made by using a Clontech In-Fusion PCR Cloning Kit (Clontech Laboratories, Palo Alto, CA) as previously described^24^.

Targeting vectors were constructed by inserting 2 kb of homologous sequences for each target site into pBluescript KS(+) by using a Clontech In-Fusion PCR Cloning Kit (Clontech Laboratories) as previously described^24^ (Supplementary Table 5). We designed targeting vectors to join two DNA sequences so that junction is the centre of predicted cleavage sites which are located within 3 bp of PAM (promoter adjacent motif) sequences^25^. For example, in the case for the *tmIn1*, the left targeting vector contained a chimeric fusion of 1 kb upstream sequence from the putative cleavage site in the *egl-4* gene and the reverse-directed 1 kb upstream sequence of the *unc-17* gene from the predicted cleavage site. The right targeting vector is composed of a chimeric fusion of the reverse-directed 1 kb downstream sequence from the putative cleavage site in the *egl-4* gene and 1 kb downstream sequence of the *unc-17* gene from the predicted cleavage site. These target vectors used together, can cause a inversion.

A Cas9 integration-site *dpy-3* genome fragment containing approximately 500 bp of sequence homologous to the target site was inserted into pPD95.79, using *EcoR*I and *BamH*I sites as previously described^16^. Plasmids for the transgenic markers *Pmyo-2::Venus* and *Pdpy-7::DsRed* were generated as previously described^26^.

### DNA microinjection

Plasmids were prepared for injection using Qiagen’s Midi Plasmid Purification Kit (QIAGEN, Hilden, Germany). The targeting vectors were linearized from purified plasmids by PCR amplification and were purified using Illustra GFX PCR DNA and a Gel Band Purification Kit (GE Healthcare, Little Chalfont, UK). To generate new balancers, the following concentrations of injection mix were used: 45 ng/μl Cas9-sgRNA #1 dual expression vector, 45 ng/μl Cas9-sgRNA #2 dual expression vector, 40 ng/μl targeting vector (left side), 40 ng/μl targeting vector (right side) and 30 ng/μl *Pmyo-2::Venus* transgene marker. To generate the integrated strain, the following concentrations of injection mix were used: 100 ng/μl Cas9-sgRNA dual expression vector and 40 ng/μl *Pdpy-7::DsRed* transgene marker. The injection mix was centrifuged for 3 min at 15,000 × g at 4 °C in Ultrafree-MC filter devices (Millipore, Massachusetts, MA). The injection mix was injected into the germ lines of adult hermaphrodite worms by using standard methods as previously described^26^. Importantly, the total Cas9-sgRNA plasmid concentration of the injection mix should be lower than 100 ng/μl. When the Cas9-sgRNA concentration exceeded 100 ng/μl, the F_1_ progeny were sterile.

### Screening for the generation of new balancers using the CRISPR/Cas9 system

To screen for new genetic balancers, injected P_0_ worms were grown on NGM plates at 20 °C for three days. We picked fluorescent F_1_ worms to individual plates at 20 °C (for example, Table 1 F_1_ worms). First screening: after three days, we selected plates which contained phenotype-positive F_2_ worms (for example, Table 1 phenotype-positive worms). By this way, we chose the F_1_ worms whose genome was cut by Cas9 at the target sites. We then picked F_1_ worms to lysis buffer (500 μg/ml proteinase K, 100 mM NaCl, 50 mM Tris, 20 mM EDTA, and 1% SDS) and confirmed by nested-PCR amplification with primers (Supplementary Table 5), whose sequences are not included in the targeting vectors (for example, Table 1 F_1_ PCR). Second screening: To examine whether the rearrangements occurred in the germline of the animals and they were heritable, we then picked F_2_ animals and performed the same PCR as above (for example, Table 1 F_2_ PCR). We isolated positive bands and determined and aligned the sequences of both breakpoints. After we identified strains with heritable rearrangements, we singled their F_2_ progeny to individual plates at 20 °C and cultured them for three days and confirmed the presence of the phenotype-positive F_3_ in the plates.

### Test for balancer chromosome

The *tmIn3*(*IV*) rearrangement was chosen to examine whether it could balance a recessive lethal mutation. Heterozygous *tmIn3*/+ hermaphrodites were mated with heterozygous *lin-1*/+ males. The F_1_ progeny from each cross plate were transferred to individual plates at 20 °C for three days. After self-fertilization, the *lin-1/tmIn3* hermaphrodites produced offspring that segregated into three genotypes, *lin-1/tmIn3*, *tmIn3/tmIn3* and *lin-1/lin-1*, and were distinguishable according to their phenotypes.

The *tmIn4*(*II*) rearrangement was also examined to determine whether it could balance a recessive lethal mutation. Heterozygous *tmIn4*/+ hermaphrodites were mated with heterozygous *mlt-7*/+ males. The F_1_ progeny from each cross plate were transferred to individual plates at 20 °C for three days. After self-fertilization, *mlt-7/tmIn4* hermaphrodites produced offspring segregating into three genotypes, *mlt-7/tmIn4*, *tmIn4/tmIn4* and *mlt-7/mlt-7*, which were distinguishable according to their phenotypes.

### Whole-genome sequencing

Genomic DNA was extracted from starved worms. Fragmentation of the genome into approximately 140 bp segments and preparation of genomic libraries were performed using automated Library Builder system (Thermo Fisher Scientific). Then, sequence templates were synthesised from the prepared libraries using the Ion Chef system, and the templates were sequenced by Ion Proton (Thermo Fisher Scientific, Massachusetts, MA) according to standard protocols (https://ioncommunity.thermofisher.com/docs/DOC-8775).

### Detection of structural variants

Raw sequencing reads were primarily mapped to the reference sequence by using TMAP software (https://github.com/iontorrent/TMAP). The reference sequence was prepared by adding sequences of *Pmyo-2::Venus*, pDD162 (Addgene plasmid 47549) and pBlueScript II KS(+) to the *C. elegans* genome sequence (ftp://ftp.wormbase.org/pub/wormbase/species/c_elegans/sequence/genomic). After primary mapping, we calculated the mean value for all read lengths. The product of the average read length times the number of reads was divided by the length of the reference sequence. The result was defined as the coverage (Supplementary Table 1). Then, the genomic rearrangements were detected by following processes.

From the primary mapping results, we obtained clipped reads, which contained both mapped and unmapped sequences (Supplemental Fig. 6a, solid and broken lines). We selected unmapped sequences that were longer than 20 bp and extracted all the continuous 16-base sequences from the unmapped reads and their complementary sequences as queries for the following realignment. From the reference sequence, the regions that perfectly matched the queries were searched by using the Aho-Corasick algorithm^27^. Then, the unmapped sequences were compared and aligned to the neighbouring sequences of each matched regions using the Smith–Waterman algorithm^28^. For the algorithm, the values used for matching, mismatching and gap score were +2, −1 and −2, respectively. Through this alignment, we detected the most homologous regions for each unmapped sequence. If there was more than one candidate for the most homologous region of an unmapped sequence, we selected the one that was nearest to the mapped region of the original clipped read.

As a result, we obtained split reads (SR) whose 5′- and 3′-regions were mapped to different sites of the reference^29^. Next, the split reads were classified into the following 5 categories: deletion-, insertion-, inversion-, translocation- and translocational inversion-type SR. When the 3′-region of an SR was aligned downstream or upstream of the site where the 5′-region of the read was mapped, the SR was defined as a deletion- or insertion-type SR. Otherwise, when the 5′-region of an SR was aligned to the reverse strand of the 3′-region of the read in the same linkage group, the SR was defined as an inversion-type SR. If the 5′- and 3′-regions of an SR were aligned to different linkage groups, the read was defined as a translocation-type SR. If an SR was determined to be both translocation and inversion-type, the SR was defined as a translocational inversion-type SR (Supplementary Fig. 6a, middle panel). After classification, we eliminated SRs that were also detected in control data. If the number of deletion-type SRs that contained a common gap between the 5′- and 3′-regions was greater than 2, we defined the region as a deletion candidate. Additionally, we investigated the combination of two types of SRs to detect complicated variant candidates. When combined deletion- and insertion-type SRs were located on both sides of a region, the region was defined as an insertion candidate. Similarly, when two inversion-type SRs were located on both sides of a region, the region was defined as a local inversion or inverted insertion candidate. The translocated insertion and inverted translocational insertion candidates were also defined using two translocation- and translocational inversion-type SRs. If there were gaps near the border of a variant region, the variant was also defined as a deletion candidate (Supplementary Fig. 6a, lower panel). To improve the reliability, complicated variants were removed when fewer than ten reads contained common variant regions.

We also counted the number of reads covering each base of the reference sequence as the depth of sequence. Regions in which the depth values were greater than one were defined as mapped regions (Supplementary Table 1). In mapped regions, the depth values were divided by the coverage value, and the quotient was defined as the normalized depth (ND) (Supplementary Fig. 6b, left panel). Then, the ratio of the ND between the balanced strains and *tmIn3* was calculated as the depth ratio (DR) value (Supplementary Fig. 6b, right panel). A low DR value meant that the copy number of the base was lower than that in the control, thus suggesting that the base was deleted in the balanced strain. Finally, we evaluated the variant candidates investigated by SR analysis using DR values. When the DR value of a deleted region was higher than 0.75, the variant was removed. Furthermore, when the DR value of insertion variants was greater than 1.75 or 2.5, the variants were defined as duplications or multiplications, respectively.

### Generation of the *tmC1;tmEx4487* transgenic line

To generate *tmC1;tmEx4487* transgenic worms, 20 ng/μl Cas9 integration-site *dpy-3* genome fragment, 160 ng/μl *Pmyo-2::Venus* and 20 ng/μl *Punc-18::unc-18 (unc-18* rescue construct) were co-injected into *tmC1* worms by using standard methods as previously described^30^.

### Generation of integrated strains by using the CRISPR/Cas9 system

Integration of extrachromosomal arrays into a balancer line was performed as previously described^16^. To screen for integrated strains, we first removed the *lig-4 (tm750*) background, and the injected P_0_ *tmC1;tmEx4487* worms were grown on NGM plates at 20 °C for four days. After self-fertilization, we picked F_1_ worms with Venus and DsRed fluorescence and transferred them to individual plates, where they were incubated at 20 °C for four days. If their F_2_ progeny carried integrated *Pmyo-2::Venus* constructs in *tmC1*, Dpy progeny would express Venus in the pharynx. In contrast, in F_2_ progeny carrying only *tmEx4487*, Dpy progeny would not express Venus. To confirm integration, F_2_ Dpy animals were transferred to individual plates and grown at 20 °C for four days. After self-fertilization, if the F_3_ Dpy progeny carried the desired integration, all Dpy progeny would express Venus.

## Additional Information

**How to cite this article**: Iwata, S. *et al.* Engineering new balancer chromosomes in *C. elegans* via CRISPR/Cas9. *Sci. Rep.* **6**, 33840; doi: 10.1038/srep33840 (2016).

## Supplementary Material

Supplementary Information

## Figures and Tables

**Table 1:** Summary of experimental efficiencies to generate the genetic balancers IV.

Balancer name	Cas9 targets	Targeting vector	Distance (cM)	Background genotype	P_0_ worms^a^	F_1_ worms^b^	Phenotype in F_2_^c^	F_1_ PCR^d^	F_2_ PCR^e^	Ratio (%)^f^
*tmIn42-44*	*egl-4 unc-17*	−	11.5	WT (N2)	146	900	163	4	3	0.33
*tmIn45*	*egl-4 unc-17*	−	11.5	*lig-4 (tm750*)	149	723	87	10	1	0.13
*−*	*egl-4 unc-17*	+	11.5	WT (N2)	107	755	102	12	0	0
*tmIn1*	*egl-4 unc-17*	+	11.5	*lig-4 (tm750*)	31	136	40	6	1	0.73
*tmIn2*	*ced-2 unc-17*	+	13.5	*lig-4 (tm750*)	48	168	24	2	1	0.60
*tmIn3*	*jtr-1 unc-17*	+	16.4	*lig-4 (tm750*)	46	312	96	25	1	0.32
*−*	*csn-4 egl-4*	+	7.6	*lig-4 (tm750*)	39	168	64	4	0	0

^a^Total number of injected P_0_ worms.

^b^Total number of fluorescent F_1_ worms obtained.

^c^Number of F_1_ strains whose progeny showed phenotypes.

^d^Number of F_1_ strains that showed rearrangement-specific PCR bands in the first screening.

^e^Number of F_2_ strains that showed rearrangement-specific PCR bands in the second screening.

^f^Isolated genetic balancer/total number of fluorescent F_1_ worms.
