# Supplementary material for: Engineering new balancer chromosomes in C. elegans via CRISPR/Cas9
Source: Sci Rep. 2016 Sep 21;6:33840. doi: 10.1038/srep33840 (PMC5030659; doi:10.1038/srep33840)
Supplement: Supplementary Information [file srep33840-s1.pdf]

Supplementary Information (Iwata et al.)

Engineering new balancer chromosomes in *C. elegans* via CRISPR/Cas9

Satoru Iwata[1], Sawako Yoshina[1], Yuji Suehiro[1], Sayaka Hori[1], Shohei Mitani[1, 2, #]

[1]Department of Physiology, Tokyo Women's Medical University School of Medicine, Tokyo, Japan

[2]Tokyo Women's Medical University Institute for Integrated Medical Sciences

[#]To whom correspondence should be addressed.

Supplementary Fig. 1: Map of *C. elegans* genome coverage by available balancers and rearrangements. Chromosomal regions shown by grey boxes are covered by genetic rearrangements such as translocations, inversions and crossover-suppressors from previous studies. The black boxes represent uncovered regions. Balancers generated in this study are indicated as red boxes. Abbreviations: *T* (translocation), *In* (inversion), and *C* (crossover-suppressor).

Supplementary Fig. 2: Schematic design for creating genome rearrangements by using the CRISPR/Cas9 system. Expressed Cas9 induces DSBs on a chromosome at locations determined by the two sgRNAs. The DSBs are repaired using HR with the targeting vectors or by other mechanisms, leading to genomic rearrangements. a (yellow), b (green), c (striped pattern), d (blue), and e (orange) boxes are used to show

how chromosomal regions are cut and joined.

Supplementary Fig. 3: Chromosomal inversion *tmIn2* between *ced-2* and *unc-17* induced by the CRISPR/Cas9 system. (**a**) Schematic of the genetic rearrangement *tmIn2*. *tmIn2* resulted from an inversion between *ced-2* and *unc-17*. (**b**) PCR amplification of the breakpoint junctions in wild-type (WT) and *tmIn2* animals. (**c**) Breakpoint sequence alignments for the WT, targeting vector and *tmIn2* alleles. Black bars indicate the predicted cleavage sites.

Supplementary Fig. 4: Chromosomal inversion *tmIn3* between *jtr-1* and *unc-17* induced by the CRISPR/Cas9 system. (**a**) Schematic of the genetic rearrangement *tmIn3*. *tmIn3* resulted from an inversion between *jtr-1* and *unc-17*. (**b**) PCR amplification of the breakpoint junctions in WT and *tmIn3* animals. (**c**) Breakpoint sequence alignments for the WT, targeting vector and *tmIn3* alleles. Black bars indicate the predicted cleavage sites.

Supplementary Fig. 5: Chromosomal inversion *tmIn3* can balance a lethal mutation. (**a**) An overview of the animal crossing schemes used to test whether *tmIn3* could balance a *lin-1* mutation. (**b**) The *lin-1/tmIn3* worms were phenotypically normal. (**c**) Genotyping of 16 animals was performed to confirm the heterozygosity of *lin-1* and the balancer *tmIn3*. (**d**) The *lin-1/lin-1* worms had a lethal phenotype. (**e**) Genotyping of 16 animals confirming the homozygosity of *lin-1* and the absence of the balancer *tmIn3*.

(**f**) The *tmIn3/tmIn3* worms showed a larval arrest phenotype. (**g**) Genotypingof 16 animals confirming the presence of *lin-1*(+/+) and the absence of wild-type *jtr-1* and *unc-17*. Scale bars represent 100 μm.

Supplementary Fig. 6: Structural variants detected by split read and copy number analysis. (**a**) According to the realignment, clipped reads were classified into 5 types (middle panel), and candidate regions of structural variants were identified by a combination of the classified reads (lower panel). (**b**) Low and high copy number regions were determined according to the ratio of normalized depth between sample and control data. (**c**) Finally, the deleted and multiplicated regions were selected by testing whether the candidate region corresponded to low and high copy regions, respectively.

Supplementary Fig. 7: Chromosomal inversion *tmIn4* between *lin-8* and *dpy-2* induced by the CRISPR/Cas9 system. (**a**) Schematic of the genetic rearrangement *tmIn4*. *tmIn4* resulted from an inversion between *lin-8* and *dpy-2*. (**b**) PCR amplification of the breakpoint junctions in WT and *tmIn4* animals. (**c**) Breakpoint sequence alignments of WT, targeting vector and *tmIn4*. Black bars indicate the predicted cleavage sites. (**d**) The relative position of breakpoints on chromosomal balancer II. The generated balancer is indicated by red double-headed arrows. Failures in the isolation of inversions are shown by white arrows with cross marks. (**e**) *tmIn4* animals exhibited a recessive dumpy (Dpy) phenotype. Scale bars represent 100 μm.

Supplementary Fig. 8: Usage of chromosomal inversion *tmIn4* to balance a lethal mutation. (**a**) Phenotypes and genotypes resulting from the self-fertilization of *mlt-7/tmIn4* heterozygotes. The *mlt-7/tmIn4* worms were phenotypically normal. (**b**) Genotyping of 16 animals was performed to determine the presence of the *mlt-7* heterozygote and the balancer *tmIn4* heterozygote. (**c**) The *mlt-7/mlt-7* worms had a lethal phenotype. (**d**) Genotyping of 16 animals was performed to determine the presence of the *mlt-7* homozygous deletion and the absence of the balancer *tmIn4*. (**e**) The *tmIn4/tmIn4* worms exhibited the dumpy (Dpy) phenotype. (**f**) Genotyping of 16 animals was performed to determine the presence of *mlt-7*(+/+) and the absence of *lin-8* and *dpy-2*. Scale bars represent 100 μm.

Supplementary Fig. 9: Chromosomal translocation *tmT3* between *pal-1(III)* and *unc-17(IV)* induced by the CRISPR/Cas9 system. (**a**) Schematic of the genetic rearrangement *tmT3*. *tmT3* resulted from a translocation between *pal-1(III)* and *unc-17(IV)*. (**b**) PCR detection of the breakpoint junctions in wild-type (WT) and *tmT3* animals. (**c**) Breakpoint sequence alignments of WT, targeting vector and *tmT3*. Black bars indicate the predicted cleavage sites. (**d**) Summary of experimental efficiencies to generate the chromosomal translocation.

Supplementary Fig. 10: Experimental methods to generate multi-copy integration strains. (**a**) Schematic design showing how integrated strains were engineered with the CRISPR/Cas9 system. P_0 *tmC1;tmEx4487*[*dpy-3*, *Pmyo-2::Venus, Punc-18::unc-18*]

worms were co-injected with a Cas9/sgRNA dual expression vector and a DsRed transgene marker. Fluorescent F_3 dumpy (Dpy) progeny carrying the balancer chromosome contained the dominant marker *Pmyo-2::Venus*. (**b**) PCR of the breakpoint junctions in WT and the integration strains. (**c**) Images of Venus localization in the *Pmyo-2::Venus* integrated strains. Images of *tmC1[tmIs1171]* and *tmC1[tmIs1172]* animals are shown by DIC and fluorescence microscopy. Scale bars represent 100 µm. (**d**) Summary of the integration efficiency to generate the integrated strain.

Supplementary Figure 11: Breakpoint sequence alignments for the WT and *tmIn42-44* alleles. Red triangles indicate cleavage sites. Sequences around breakpoints are shown by blue letters for *unc-17* and magenta letters for *egl-4*. Insertion sequences are shown by black letters. Black bars indicate the predicted cleavage sites.

Supplementary Table 1: Comparison of sequence coverage between N2 (Control) and *tm5929/tmIn3*

Sample	Read length (bp)	Coverage	Mapped region (%)
N2 (Control)	153.18	56.94	99.91
tm5929/tmIn3	141.72	39.57	99.88

Supplementary Table 2: Summary of experimental efficiencies to generate the genetic balancer II

Balancer name	Cas9 targets	Distance (cM)	Background genotype	P_0 worms[a]	F_1 worms[b]	Phenotype in F_2[c]	F_1 PCR[d]	F_2 PCR[e]	Ratio (%)[f]
-	*emc-1 lin-31*	9.6	*lig-4* (*tm750*)	41	94	35	10	0	0
-	*sup-9 lin-31*	6.3	*lig-4* (*tm750*)	39	214	35	16	0	0
-	*sqv-2 dpy-2*	9.5	*lig-4* (*tm750*)	73	257	80	2	0	0
tmIn4	*lin-8 dpy-2*	8.6	*lig-4* (*tm750*)	51	267	108	9	1	0.37

[a]total number of injected P_0 worms, [b]total number of fluorescent F_1 worms obtained, [c]number of F_1 strains whose progeny showed phenotypes, [d]number of F_1 strains that showed rearrangement-specific PCR bands in the first screening, [e]number of F_2 strains that showed rearrangement-specific PCR bands in the second screening, [f]ratios of isolated genetic balancer strains over total number of fluorescent F_1 worms.

Supplementary Table 3: Summary of experimental efficiencies to generate the genetic balancers X

Balancer name	Cas9 targets	Distance (cM)	genotype	P_0 worms	F_1 worms[a]	Phenotype in F_2	F_1 PCR[b]	F_2 PCR[c]	Inversion interval (cM)	Ratio (%)[d]
tmIn26-27	*lon-2*<br>*mec-10*	6.7	*lig-4* (*tm750*)	49	69	43	2	2	6.7	4.3
tmC1-2	*F53B1.2*<br>*unc-18*	17	*lig-4* (*tm750*)	27	184	46	8	2	11.7	1.1

[a]total number of injected P_0 worms, [b]total number of fluorescent F_1 worms obtained, [c]number of F_1 strains whose progeny showed phenotypes, [d]number of F_1 strains that showed rearrangement-specific PCR bands in the first screening, [e]number of F_2 strains that showed rearrangement-specific PCR bands in the second screening, [f]ratios of isolated genetic balancer strains over total number of fluorescent F_1 worms.

Supplementary Table 4: Summary of the break point sequence analysis

	left breakpoint			right breakpoint		
Rearrangement	left side deletion	insertion	right side deletion	left side deletion	insertion	right side deletion
tmIn1	3	25	(-)	1	24	1
tmIn2	3	26	5	1	1	4
tmIn3	3	0	1	1	(-)	1
tmIn4	12	21	1	(-)	18	1
tmIn26	(-)	(-)	(-)	(-)	(-)	(-)
tmIn27	5	(-)	10	55	(-)	6
tmC1	(-)	25	1	(-)	6	1
tmC2	6	33	18	4	(-)	3
tmT3	(-)	(-)	(-)	751	(-)	3
tmIn42	3	12	1	(-)	14	1
tmIn43	5	(-)	1	5	1	1
tmIn44	1	43	1	(-)	36	1
tmIn45	(-)	33	2	16	(-)	16

The sequence identity or indel characteristics are shown for two break points of the genome for each rearrangement; left breakpoint and right breakpoint for all the rearrangements except *tmT3*, for which left breakpoint column means the junction of III; IV and right

breakpoint column means the junction of IV; III. Each breakpoint is divided to two parts: left side and right side from putative DSB sites. Identical sequences to targeting vectors (or original genome) are indicated by “complete copy”, whereas deletions are shown by ”del” with the length in the following parenthesis, and insertions shown by “ins” with the length in the following parenthesis.

Supplementary Table 5: List of all primers used in this study

sgRNA primers

unc-17 (IV) sgRNA F	5’ -AGACTCGGAGATCCTCAAAGGTTTTAGAGCTAGAAATAGCAAGT-3’
unc-17 (IV) sgRNA R	5’ -CTTTGAGGATCTCCGAGTCTCAAGACATCTCGCAATAGG-3’
egl-4 (IV) sgRNA F	5’ -ATTGTCGACGACTTCCGAGGTTTTAGAGCTAGAAATAGCAAGT-3’
egl-4 (IV) sgRNA R	5’ -CTCGGAAGTCGTCGACAATCAAGACATCTCGCAATAGG-3’
ced-2 (IV) sgRNA F	5’ –TCCAACGGAATGTACAAAGGTTTTAGAGCTAGAAATAGCAAGT-3’
ced-2 (IV) sgRNA R	5’ -CTTTGTACATTCCGTTGGACAAGACATCTCGCAATAGG-3’
jtr-1 (IV) sgRNA F	5’ -ACGGAACACGGCGGAACCACGTTTTAGAGCTAGAAATAGCAAGT-3’
jtr-1 (IV) sgRNA R	5’ -GTGGTTCCGCCGTGTTCCGTCAAGACATCTCGCAATAGG-3’
csn-4 (IV) sgRNA F	5’ -AACATGGAGAATCTATTCTGAAGTTTTAGAGCTAGAAATAGCAAGT-3’
csn-4 (IV) sgRNA R	5’ -TTCAGAATAGATTCTCCATGTTCAAGACATCTCGCAATAGG-3’
dpy-2 (II) sgRNA F	5’ -GAAGTCCAGAGTCTCCTGAGTTTTAGAGCTAGAAATAGCAAGT-3’
dpy-2 (II) sgRNA R	5’ -TCAGGAGACTCTGGACTTCCAAGACATCTCGCAATAGG-3’
lin-8 (II) sgRNA F	5’ -CTTTCTCACAGCAGTACGGGTTTTAGAGCTAGAAATAGCAAGT-3’
lin-8 (II) sgRNA R	5’ -CCGTACTGCTGTGAGAAAGCAAGACATCTCGCAATAGG-3’
sqv-2 (II) sgRNA F	5’ -CAACACCTTCAGCCATCAAGTTTTAGAGCTAGAAATAGCAAGT-3’
sqv-2 (II) sgRNA R	5’ -TTGATGGCTGAAGGTGTTGCAAGACATCTCGCAATAGG-3’

lin-31 (II) sgRNA F	5’ -AATGACAAGTTGTGTCGCAGTTTTAGAGCTAGAAATAGCAAGT-3’
lin-31 (II) sgRNA R	5’ -TGCGACACAACTTGTCATTCAAGACATCTCGCAATAGG-3’
sup-9 (II) sgRNA F	5’ -AACACCGAAGATGAACGGAGTTTTAGAGCTAGAAATAGCAAGT-3’
sup-9 (II) sgRNA R	5’ -TCCGTTCATCTTCGGTGTTCAAGACATCTCGCAATAGG-3’
emc-1 (II) sgRNA F	5’ -CCTCTCTGGTGTTGAATACGTTTTAGAGCTAGAAATAGCAAGT-3’
emc-1 (II) sgRNA R	5’ -GTATTCAACACCAGAGAGGCAAGACATCTCGCAATAGG-3’
lon-2 (X) sgRNA F	5’ -GGAAACTATACCCTCACTGGTTTTAGAGCTAGAAATAGCAAGT-3’
lon-2 (X) sgRNA R	5’ -CAGTGAGGGTATAGTTTCCCAAGACATCTCGCAATAGG-3’
mec-10 (X) sgRNA F	5’ -TAGATTACCTGCGCCGTATGTTTTAGAGCTAGAAATAGCAAGT-3’
mec-10 (X) sgRNA R	5’ -ATACGGCGCAGGTAATCTACAAGACATCTCGCAATAGG-3’
F53B1.2 (X) sgRNA F	5’ -GCTAGTGCTTGCGGTTGTCGTTTTAGAGCTAGAAATAGCAAGT-3’
F53B1.2 (X) sgRNA R	5’ -GACAACCGCAAGCACTAGCCAAGACATCTCGCAATAGG-3’
unc-18 (X) sgRNA F	5’ -GTCAGTGGCACAAGGAACGGTTTTAGAGCTAGAAATAGCAAGT-3’
unc-18 (X) sgRNA R	5’ -CGTTCCTTGTGCCACTGACCAAGACATCTCGCAATAGG-3’
pal-1 (III) sgRNA F	5’ -GAAGTAGCAGTAGTGATAGGTTTTAGAGCTAGAAATAGCAAGT-3’
pal-1 (III) sgRNA R	5’ -CTATCACTACTGCTACTTCCAAGACATCTCGCAATAGG-3’
dpy-3 (X) sgRNA F	5’ -TCACCGTCCAGTCTGCTACGTTTTAGAGCTAGAAATAGCAAGT-3’
dpy-3 (X) sgRNA R	5’ -GTAGCAGACTGGACGGTGACAAGACATCTCGCAATAGG-3’

Targeting vector primers	
pBluescript KS(+) F	5’ -CTTATCGATACCGTCGACCTC-3’
pBluescript KS(+) R	5’ -CTTGATATCGAATTCCTGCAGC-3’
egl-4 (IV) tv_Left F	5’ -GAATTCGATATCAAGCTTTGTCACGTTCGAAATCG-3’
egl-4 (IV) tv_Left R	5’ -CGGAAGTCGTCGACAATCTTC-3’
unc-17 (IV) tv_Right F	5’ -TGTCGACGACTTCCGAGCGGACGCCAAAAAGTGGC-3’
unc-17 (IV) tv_Right R	5’ -GACGGTATCGATAAGTGTTCTGAAGCCATTTGGAA-3’
egl-4 (IV) tv F	5’ -CTTTGTCACGTTCGAAATCG-3’
unc-17 (IV) tv R	5’ -TGTTCTGAAGCCATTTGGAA-3’
unc-17 (IV) tv_Left F	5’ -GAATTCGATATCAAGGGCGCCAGTTTCAAAACATA-3’
unc-17 (IV) tv_Left R	5’ -TGTGCAAACTCCTCTTTGAGGATCTCCGAGTCTCG-3’
egl-4 (IV) tv_Right F	5’ -AGAGGAGTTTGCACAGGTTAC-3’
egl-4 (IV) tv_Right R	5’ -GACGGTATCGATAAGCCCGCCTCTTTCATGGAC-3’
unc-17 (IV) tv F	5’ -GGCGCCAGTTTCAAAACATA-3’
egl-4 (IV) tv R	5’ -CCCGCCTCTTTCATGGAC-3’
ced-2 (IV) tv_Left F	5’ -GAATTCGATATCAAGGCGCCAGTCAATCGTCGATC-3’
ced-2 (IV) tv_Left R	5’ -TTTTTGGCGTCCGCTTTGTACATTCCGTTGGACATTTTC-3’

unc-17 (IV) tv_Right F2	5’ -AGCGGACGCCAAAAAGTGGC-3’
unc-17 (IV) tv_Right R	5’ -GACGGTATCGATAAGTGTTCTGAAGCCATTTGGAA-3’
ced-2 (IV) tv F	5’ -CTTTGTCACGTTCGAAATCG-3’
unc-17 (IV) tv R	5’ -TGTTCTGAAGCCATTTGGAA-3’
unc-17 (IV) tv_Left F	5’ -GAATTCGATATCAAGGGCGCCAGTTTCAAAACATA-3’
unc-17 (IV) tv_Left R2	5’ -TTGAGGATCTCCGAGTCTCG-3’
ced-2 (IV) tv_Right F	5’ -CTCGGAGATCCTCAAAGCGGAACTCGACGGCC-3’
ced-2 (IV) tv_Right R	5’ -GACGGTATCGATAAGACATTTCTAGGCGGGATCTC-3’
unc-17 (IV) tv F	5’ -GGCGCCAGTTTCAAAACATA-3’
ced-2 (IV) tv R	5’ -ACATTTCTAGGCGGGATCTC-3’
jtr-1 (IV) tv_Left F	5’ -GAATTCGATATCAAGTCGACGGGAATGGGACGAC-3’
jtr-1 (IV) tv_Left R	5’ -TTTTTGGCGTCCGCTGGTTCCGCCGTGTTCCG-3’
unc-17 (IV) tv_Right F2	5’ -AGCGGACGCCAAAAAGTGGC-3’
unc-17 (IV) tv_Right R	5’ -GACGGTATCGATAAGTGTTCTGAAGCCATTTGGAA-3’
jtr-1 (IV) tv F	5’ -TCGACGGGAATGGGACGAC-3’
unc-17 (IV) tv R	5’ -TGTTCTGAAGCCATTTGGAA-3’
unc-17 (IV) tv_Left F	5’ -GAATTCGATATCAAGGGCGCCAGTTTCAAAACATA-3’
unc-17 (IV) tv_Left R2	5’ -TTGAGGATCTCCGAGTCTCG-3’

jtr-1 (IV) tv_Right F	5' -CTCGGAGATCCTCAAACCGGAAAACTGGAAAAAGTGG-3'
jtr-1 (IV) tv_Right R	5' -GACGGTATCGATAAGGTCGATTGCCTCGCCTGC-3'
unc-17 (IV) tv F	5' -GGCGCCAGTTTCAAAACATA-3'
jtr-1 (IV) tv R	5' -GTCGATTGCCTCGCCTGC-3'
egl-4 (IV) tv_Left F2	5' -GAATTCGATATCAAGTACAGAGAATTCGGACAGCTG-3'
egl-4 (IV) tv_Left R2	5' -GGAGAATCTATTCTGGAGAGGAGTTTGCACAGGTTACACTG-3'
unc-17 (IV) tv_Right F	5' -TGTCGACGACTTCCGAGCGGACGCCAAAAAGTGGC-3'
csn-4 (IV) tv_Right R	5' -CAGAATAGATTCTCCATGTTCATCAG-3'
egl-4 (IV) tv F2	5' -TACAGAGAATTCGGACAGCTG-3'
csn-4 (IV) tv R	5' -TCTGGAGATTATCCTGCTTTTTAAAGC-3'
csn-4 (IV) tv_Left F	5' -GAATTCGATATCAAGACTTTAAGAAAATTTTCGGACTTCCAGC-3'
csn-4 (IV) tv_Left R	5' -AAAGGAGTTATTCAAGAGCATAATATTACTG-3'
egl-4 (IV) tv_Right F2	5' -TTGAATAACTCCTTTGGAAGTCGTCGACAATCTTCAC-3'
egl-4 (IV) tv_Right R2	5' -GACGGTATCGATAAGGTCTTTGTCACGTTCGAAATCG-3'
csn-4 (IV) tv F	5' -AAAGCTGTAAGCCGCTGTG-3'
egl-4 (IV) tv R2	5' -GTCTTTGTCACGTTCGAAATCG-3'
dpy-2 (II) tv_Left F	5' -GAATTCGATATCAAGCCTTTCTTATCTCCGCCGAC-3'
dpy-2 (II) tv_Left R	5' -GATGGTCCACGTTCTCCCG-3'

lin-8 (II) tv_Right F	5’ -AGAACGTGGACCATCGTACTGCTGTGAGAAAGCGA-3’
lin-8 (II) tv_Right R	5’ -GACGGTATCGATAAGGGATTTTGGTGCAGCTCGAC-3’
dpy-2 (II) tv F	5’ -CGCATTTTGAATGAGAAGTCA-3’
lin-8 (II) tv R	5’ -GGATTTTGGTGCAGCTCGAC-3’
lin-8 (II) tv_Left F	5’ -GAATTCGATATCAAGGACCTTGTGCGTTGAGGAAC-3’
lin-8 (II) tv_Left R	5’ -AGTCCAGAGTCTCCTGGTGGCGGCGGGTCCCC-3’
dpy-2 (II) tv_Right F	5’ -AGGAGACTCTGGACTTCCAG-3’
dpy-2 (II) tv_Right R	5’ -GACGGTATCGATAAGGAAACAGTTACGGACGTCGC-3’
lin-8 (II) tv F	5’ -GACCTTGTGCGTTGAGGAAC-3’
dpy-2 (II) tv R	5’ -GAAACAGTTACGGACGTCGC-3’
emc-1 (II) tv_Left F	5’ -GAATTCGATATCAAGGGCACTCCAATCGAATCGG-3’
emc-1 (II) tv_Left R	5’ -CTCGAACATTCCGGAATTCAACACCAGAGAGGCC-3’
lin-31 (II) tv_Right F	5’ -TCCGGAATGTTCGAGAACGG-3’
lin-31 (II) tv_Right R	5’ -GACGGTATCGATAAGGGGTAGTACCGGGAGGAGAG-3’
emc-1 (II) tv F	5’ -GGCACTCCAATCGAATCGG-3’
lin-31 (II) tv R	5’ -GGGTAGTACCGGGAGGAGAG-3’
lin-31 (II) tv_Left F	5’ -GAATTCGATATCAAGAGGTATGAGTTGGGGGTTTG-3’
lin-31 (II) tv_Left R	5’ -GGCATTTGGGTGCACCG-3’

emc-1 (II) tv_Right F	5’ -GTGCACCCAAATGCCACCGGCAATATTGGTGAGTTTTTG-3’
emc-1 (II) tv_Right R	5’ -GACGGTATCGATAAGTTCTTGGACGTGACAGCTGG-3’
lin-31 (II) tv F	5’ -AGGTATGAGTTGGGGGTTTG-3’
emc-1 (II) tv R	5’ -TTCTTGGACGTGACAGCTGG-3’
sup-9 (II) tv_Left F	5’ -GAATTCGATATCAAGCCCGCTAGCAAGAGAGTGTG-3’
sup-9 (II) tv_Left R	5’ -CTCGAACATTCCGGACGTTCATCTTCGGTGTTCATAGTC-3’
lin-31 (II) tv_Right F	5’ -TCCGGAATGTTCGAGAACGG-3’
lin-31 (II) tv_Right R	5’ -GACGGTATCGATAAGGGGTAGTACCGGGAGGAGAG-3’
sup-9 (II) tv F	5’ -CCCGCTAGCAAGAGAGTGTG-3’
lin-31 (II) tv R	5’ -GGGTAGTACCGGGAGGAGAG-3’
lin-31 (II) tv_Left F	5’ -GAATTCGATATCAAGAGGTATGAGTTGGGGGTTTG-3’
lin-31 (II) tv_Left R	5’ -GGCATTTGGGTGCACCG-3’
sup-9 (II) tv_Right F	5’ -GTGCACCCAAATGCCGAGGGATGAACAGTGAGTTTTTTCTG-3’
sup-9 (II) tv_Right R	5’ -GACGGTATCGATAAGTCTCCTTGTTGCCCGAAATC-3’
lin-31 (II) tv F	5’ -AGGTATGAGTTGGGGGTTTG-3’
sup-9 (II) tv R	5’ -TCTCCTTGTTGCCCGAAATC-3’
dpy-2 (II) tv_Left F	5’ -GAATTCGATATCAAGCCTTTCTTATCTCCGCCGAC-3’
dpy-2 (II) tv_Left R	5’ -GATGGTCCACGTTCTCCCG-3’

sqv-2 (II) tv_Right F	5’ -AATGGTTCGGAGCAATTTTAAGC-3’
sqv-2 (II) tv_Right R	5’ -GACGGTATCGATAAGTTTGACGTCGTTCTGTTTCG-3’
dpy-2 (II) tv F	5’ -CGCATTTTGAATGAGAAGTCA-3’
sqv-2 (II) tv R	5’ -TTTGACGTCGTTCTGTTTCG-3’
sqv-2 (II) tv_Left F	5’ -GAATTCGATATCAAGCGTATTTCCACGTGTCTATCCG-3’
sqv-2 (II) tv_Left R	5’ -AGTCCAGAGTCTCCTGATGGCTGAAGGTGTTGCT-3’
dpy-2 (II) tv_Right F	5’ -AGGAGACTCTGGACTTCCAG-3’
dpy-2 (II) tv_Right R	5’ -GACGGTATCGATAAGGAAACAGTTACGGACGTCGC-3’
sqv-2 (II) tv F	5’ -CGTATTTCCACGTGTCTATCCG-3’
dpy-2 (II) tv R	5’ -GAAACAGTTACGGACGTCGC-3’
lon-2 (X) tv_Left F	5’ -GAATTCGATATCAAGAGCCATTGTGCAATTGCATG-3’
lon-2 (X) tv_Left R	5’ -CTGTGGAAGTGAAAAACATGCA-3’
mec-10 (X) tv_Right F	5’ -TTTTCACTTCCACAGTATGGGGATTGTGTAGAGGATG-3’
mec-10 (X) tv_Right R	5’ -GACGGTATCGATAAGCTCCTGACCAAAGTCCAAGG-3’
lon-2 (X) tv F	5’ -AGCCATTGTGCAATTGCATG-3’
mec-10 (X) tv R	5’ -CTCCTGACCAAAGTCCAAGG-3’
mec-10 (X) tv_Left F	5’ -GAATTCGATATCAAGTGGCGATAGTGAAGATTTCTCTG-3’
mec-10 (X) tv_Left R	5’ -GAAACTATACCCTCACGGCGCAGGTAATCTACTC-3’

lon-2 (X) tv_Right F	5’ -TGAGGGTATAGTTTCCCTTTTGT-3’
lon-2 (X) tv_Right R	5’ -GACGGTATCGATAAG AGAGAGCGACAAGTGATGGG-3’
mec-10 (X) tv F	5’ -TGGCGATAGTGAAGATTTCTCTG-3’
lon-2 (X) tv R	5’ -AGAGAGCGACAAGTGATGGG-3’
F53B1.2 (X) tv_Left F	5’ -GAATTCGATATCAAGTTTGCGTTCTTGCACTCGCG-3’
F53B1.2 (X) tv_Left R	5’ -CAACCGCAAGCACTAGCCC-3’
unc-18 (X) tv_Right F	5’ -TAGTGCTTGCGGTTGCGAGGACAACAATCCAACTATCGC-3’
unc-18 (X) tv_Right R	5’ -GACGGTATCGATAAGAAATAGTTATTCCGTCGAGTTGATC-3’
F53B1.2 (X) tv F	5’ -TTTGCGTTCTTGCACTCGCG-3’
unc-18 (X) tv R	5’ -AAATAGTTATTCCGTCGAGTTGATC-3’
unc-18 (X) tv_Left F	5’ -GAATTCGATATCAAGTGGCGATAGTGAAGATTTCTCTG-3’
unc-18 (X) tv_Left R	5’ -TTCCTTGTGCCACTGACCATATC-3’
F53B1.2 (X) tv_Right F	5’ -CAGTGGCACAAGGAATCTGGTTTACTGTGCTCTTTTTCCACAA-3’
F53B1.2 (X) tv_Right R	5’ -GACGGTATCGATAAGTTCAAAGAACCACCCGAGCG-3’
unc-18 (X) tv F	5’ -TGGCGATAGTGAAGATTTCTCTG-3’
F53B1.2 (X) tv R	5’ -TTCAAAGAACCACCCGAGCG-3’
pal-1 (III) tv_Left F	5’ -GAATTCGATATCAAGGACAGTTTCCTGCACAGACC-3’
pal-1 (III) tv_Left R	5’ -TTTTTGGCGTCCGCTATCACTACTGCTACTTCCAT-3’

unc-17 (IV) tv_Right F	5’ -AGCGGACGCCAAAAAGTGGC-3’
unc-17 (IV) tv_Right R	5’ -GACGGTATCGATAAGTGTTCTGAAGCCATTTGGAA-3’
pal-1 (III) tv F	5’ -GACAGTTTCCTGCACAGACC-3’
unc-17 (IV) tv R	5’ -TGTTCTGAAGCCATTTGGAA-3’
unc-17 (IV) tv_Left F	5’ -GAATTCGATATCAAGGGCGCCAGTTTCAAAACATA-3’
unc-17 (IV) tv_Left R3	5’ -TGGATACATTCCACTTTGAGGATCTCCGAGTCTCG-3’
pal-1 (III) tv_Right F	5’ -AGTGGAATGTATCCATCACCG-3’
pal-1 (III) tv_Right R	5’ -CGGTATCGATATAGCTTCGGTATTTGTCAGCTGTTCG-3’
unc-17 (IV) tv F	5’ -GGCGCCAGTTTCAAAACATA-3’
pal-1 (III) tv R	5’ -CGGTATTTGTCAGCTGTTCG-3’

Genotyping primers

egl-4-unc-17 (IV) F	5’ -CTTTGTCACGTTCGAAATCG-3’
egl-4-unc-17 (IV) R	5’ -TAAATTGCTCGGCGAGTTG-3’
egl-4-unc-17 (IV) F2	5’ -TTGCACGAATTTTGGAGGA-3’
egl-4-unc-17 (IV) R2	5’ -TTTAATGCCAGGAATGGGGA-3’
unc-17-egl-4 (IV) F	5’ -GGCGCCAGTTTCAAAACATA-3’
unc-17-egl-4 (IV) R	5’ -AAGGCATACCTCGAGCAGC-3’

unc-17-egl-4 (IV) F2	5’ -TGGCGGGAAATAAAAGAACA-3’
unc-17-egl-4 (IV) R2	5’ -TTACAGAGAATTCGGACAGCTG-3’
ced-2-unc-17 (IV) F	5’ -GAACCAGGATTGGTGGGAGG-3’
ced-2-unc-17 (IV) R	5’ -TAAATTGCTCGGCGAGTTG-3’
ced-2-unc-17 (IV) F2	5’ -AACCACTGGATTAGTGCCGG-3’
ced-2-unc-17 (IV) R2	5’ -TTTAATGCCAGGAATGGGGA-3’
unc-17-ced-2 (IV) F	5’ -CTGACGAATATGCCGCAAAC-3’
unc-17-ced-2 (IV) R	5’ -TTTCAGAGATTCCTGGCCCG-3’
unc-17-ced-2 (IV) F2	5’ -TGGCGGGAAATAAAAGAACA-3’
unc-17-ced-2 (IV) R2	5’ -GAGAAAAATACGCCGAGCTTTC-3’
jtr-1-unc-17 (IV) F	5’ -CTCAGCCACAGATGTTCACGG-3’
jtr-1-unc-17 (IV) R	5’ -TAAATTGCTCGGCGAGTTG-3’
jtr-1-unc-17 (IV) F2	5’ -GTGTGGATAGGGCTGTGTCG-3’
jtr-1-unc-17 (IV) R2	5’ -TTTAATGCCAGGAATGGGGA-3’
unc-17-jtr-1 (IV) F	5’ -CTGACGAATATGCCGCAAAC-3’
unc-17-jtr-1 (IV) R	5’ -TCCTTACACTATGTAGTCTGTGAAG-3’
unc-17-jtr-1 (IV) F2	5’ -TGGCGGGAAATAAAAGAACA-3’
unc-17-jtr-1 (IV) R2	5’ -CTCCAGAAGTCGATTGCCT-3’

egl-4-csn-4 (IV) F	5’ -TGGCGTGACTAAATAGGCTC-3’
egl-4-csn-4 (IV) R	5’ -TTTTAAAAAAATTGTGAAATTTCGCTGAATCACG-3’
egl-4-csn-4 (IV) F2	5’ -ATTGTCCTAATAGGCTCCGC-3’
egl-4-csn-4 (IV) R2	5’ -CCTTTAAAACTCACCTAAAAGTGTGG-3’
csn-4-egl-4 (IV) F	5’ -ACTTTAAGAAAATTTTCGGACTTCCAGC-3’
csn-4-egl-4 (IV) R	5’ -TTGCAGAAATTCTTACAATTTAAAGGAG-3’
csn-4-egl-4 (IV) F2	5’ -TATTTAACTGAACATTTATGACAATTCCG-3’
csn-4-egl-4 (IV) R2	5’ -TATGTCCGAAGTTGAAGCCG-3’
dpy-2-lin-8 (II) F	5’ -TGAGTGTAATCACTACGTGACC-3’
dpy-2-lin-8 (II) R	5’ -GTGGCTCGACGCACAAGG-3’
dpy-2-lin-8 (II) F2	5’ -CTCGTCTTCTGGATCGGATAC-3’
dpy-2-lin-8 (II) R2	5’ -GATCTTTCGCTACTTTCACCG-3’
lin-8-dpy-2 (II) F	5’ -ATTGTTGATCATGGCCGGGG-3’
lin-8-dpy-2 (II) R	5’ -CGTTGTGGCCACATGTTTCG-3’
lin-8-dpy-2 (II) F2	5’ -ACCGCAGTGGGTTTATCGAC-3’
lin-8-dpy-2 (II) R2	5’ -GATGTAGTACATGACCGATATCTCC-3’
emc-1-lin-31 (II) F	5’ -TGCCCCAGTCCTAAATGGAG-3’
emc-1-lin-31 (II) R	5’ -TGCCTAAGTGCTTGCCTACA-3’

emc-1-lin-31 (II) F2	5’ -TATTTTCGCCTCGGTGACG-3’
emc-1-lin-31 (II) R2	5’ –AACTATTGCAACAGATGGCAC-3’
lin-31-emc-1 (II) F	5’ -GAGCAGAAGCCACCGTACTC-3’
lin-31-emc-1 (II) R	5’ -CAAAAGCTCCATCCTCGGTG-3’
lin-31-emc-1 (II) F2	5’ -GATAAAATGCTCCCGCTCAC-3’
lin-31-emc-1 (II) R2	5’ -CGACACGTGGATTACAACGTC-3’
sup-9-lin-31 (II) F	5’ -GCTCCGTTTCATAAGACGAGC-3’
sup-9-lin-31 (II) R	5’ -TGCCTAAGTGCTTGCCTACA-3’
sup-9-lin-31 (II) F2	5’ -GGAAACAACCAATAGTGACGTC-3’
sup-9-lin-31 (II) R2	5’ -AACTATTGCAACAGATGGCAC-3’
lin-31-sup-9 (II) F	5’ -GAGCAGAAGCCACCGTACTC-3’
lin-31-sup-9 (II) R	5’ -GAGGTACCTGGTGAGAAGAC-3’
lin-31-sup-9 (II) F2	5’ -GATAAAATGCTCCCGCTCAC-3’
lin-31-sup-9 (II) R2	5’ -GCATTATGGACTGAGATTCGTC-3’
dpy-2-sqv-2 (II) F	5’ -TGAGTGTAATCACTACGTGACC-3’
dpy-2-sqv-2 (II) R	5’ -TTCCGACGGCGAATTTTGCG-3’
dpy-2-sqv-2 (II) F2	5’ -CTCGTCTTCTGGATCGGATAC-3’
dpy-2-sqv-2 (II) R2	5’ -CGGAACCATGTGTCACGGAC-3’

sqv-2-dpy-2 (II) F	5’ -CGCCCGTATTTCCACG-3’
sqv-2-dpy-2 (II) R	5’ -CGTTGTGGCCACATGTTTCG-3’
sqv-2-dpy-2 (II) F2	5’ -CCTCTTCTCTTGTACTTGTCGAC-3’
sqv-2-dpy-2 (II) R2	5’ -GATGTAGTACATGACCGATATCTCC-3’
lon-2-mec-10 (X) F	5’ -TTTGGCTGCCCACTCTCGAG-3’
lon-2-mec-10 (X) R	5’ -TGCTGGTTTATGTGATGTGC-3’
lon-2-mec-10 (X) F2	5’ -TGTGATTCCAGCCATTGTGC-3’
lon-2-mec-10 (X) R2	5’ -CCATGTATATCAGCTCAAAGGC-3’
mec-10-lon-2 (X) F	5’ -CATGCTGTTAACTGAAGTCCTG-3’
mec-10-lon-2 (X) R	5’ -TTCCATCCGGTGAGCCCTTC-3’
mec-10-lon-2 (X) F2	5’ -TCCAGGCTGCAATGGTATTG-3’
mec-10-lon-2 (X) R2	5’ -CCCATCTTGCAATCAGTTGTTC-3’
F53B1.2-unc-18 (X) F	5’ -ATTCAGGCCTTGTGTGGCTG-3’
F53B1.2-unc-18 (X) R	5’ -GTGACATTTTGGAATATAGTCATTATTGTG-3’
F53B1.2-unc-18 (X) F2	5’ -TTCTACGTTTCCTTCAACTAATCTTC-3’
F53B1.2-unc-18 (X) R2	5’ -ACACAACGTCAAATCACAATCC-3’
unc-18-F53B1.2 (X) F	5’ -TCAAGCAATGTGCTACGACC-3’
unc-18-F53B1.2 (X) R	5’ -TTCCTTGCGATCCATATTCAAAG-3’

unc-18-F53B1.2 (X) F2	5’ -TGACGTTTACAAGTACGAAACG-3’
unc-18-F53B1.2 (X) R2	5’ -CGGGAACCAGGTCCTCATCC -3’
pal-1-unc-17 (III;IV) F	5’ -GACAGTTTCCTGCACAGACC-3’
pal-1-unc-17 (III;IV) R	5’ -TAAATTGCTCGGCGAGTTG-3’
pal-1-unc-17 (III;IV) F2	5’ -TGTCACGTGGCCTCACTATC-3’
pal-1-unc-17 (III;IV) R2	5’ -TTTAATGCCAGGAATGGGGA-3’
unc-17- pal-1 (III;IV) F	5’ -GGCGCCAGTTTCAAAACATA-3’
unc-17- pal-1 (III;IV) R	5’ -TCCACGAGAATCCCTGAAAC-3’
unc-17- pal-1 (III;IV) F2	5’ -CTGACGAATATGCCGCAAAC-3’
unc-17- pal-1 (III;IV) R2	5’ -CAGATCAAAAATTGGCTGCTT-3’
lin-1 (IV) F	5’ -CTTGGGTTTCGACATGTGTC-3’
lin-1 (IV) R	5’ -CTTAGGGCTATGCGCCTTTA-3’
jtr-1 (IV) F	5’ -CTCAGCCACAGATGTTCACGG-3’
jtr-1 (IV) R	5’ -TCCTTACACTATGTAGTCTGTGAAG-3’
jtr-1 (IV) F2	5’ -GTGTGGATAGGGCTGTGTCG-3’
jtr-1 (IV) R2	5’ -CTCCAGAAGTCGATTGCCT-3’
unc-17 (IV) F	5’ -CTGACGAATATGCCGCAAAC-3’
unc-17 (IV) R	5’ -TAAATTGCTCGGCGAGTTG-3’

unc-17 (IV) F2	5' -TGGCGGGAAATAAAAGAACA-3'
unc-17 (IV) R2	5' -TTTAATGCCAGGAATGGGGA-3'
mlt-7 (II) F	5' -CTTGAGGCCTAGGTAGGCTC-3'
mlt-7 (II) R	5' -GCCTGTGCAATACTGTCTTT-3'
lin-8 (II) F	5' -ATTGTTGATCATGGCCGGGG-3'
lin-8 (II) R	5' -GTGGCTCGACGCACAAGG-3'
lin-8 (II) F2	5' -ACCGCAGTGGGTTTATCGAC-3'
lin-8 (II) R2	5' -GATCTTTCGCTACTTTCACCG-3'
dpy-2 (II) F	5' -TGAGTGTAATCACTACGTGACC-3'
dpy-2 (II) R	5' -CGTTGTGGCCACATGTTTCG-3'
dpy-2 (II) F2	5' -CTCGTCTTCTGGATCGGATAC-3'
dpy-2 (II) R2	5' -GATGTAGTACATGACCGATATCTCC-3'
dpy-3 (X) F	5' -CTCTAATGATTGGTGACGTTGG-3'
dpy-3 (X) R	5' -CTTTTTCTGGAGTCAGATGACAG-3'
dpy-3 (X) F2	5' -CTCCAGATACGCTTAGAAAATAGC-3'
dpy-3 (X) R2	5' -TCTCGTAATGACGTAAATGTGTATG-3'

Integrated-site genome primers

dpy-3 (X) Is F	5' -CGACTCTAGAGGATCCGAACAGTGAAGTAGACTTCTGCAAG-3'
dpy-3 (X) Is R	5' -CGCTCAGTTGGAATTCCATCTTGTCCTGATGTACCGGCAT-3'

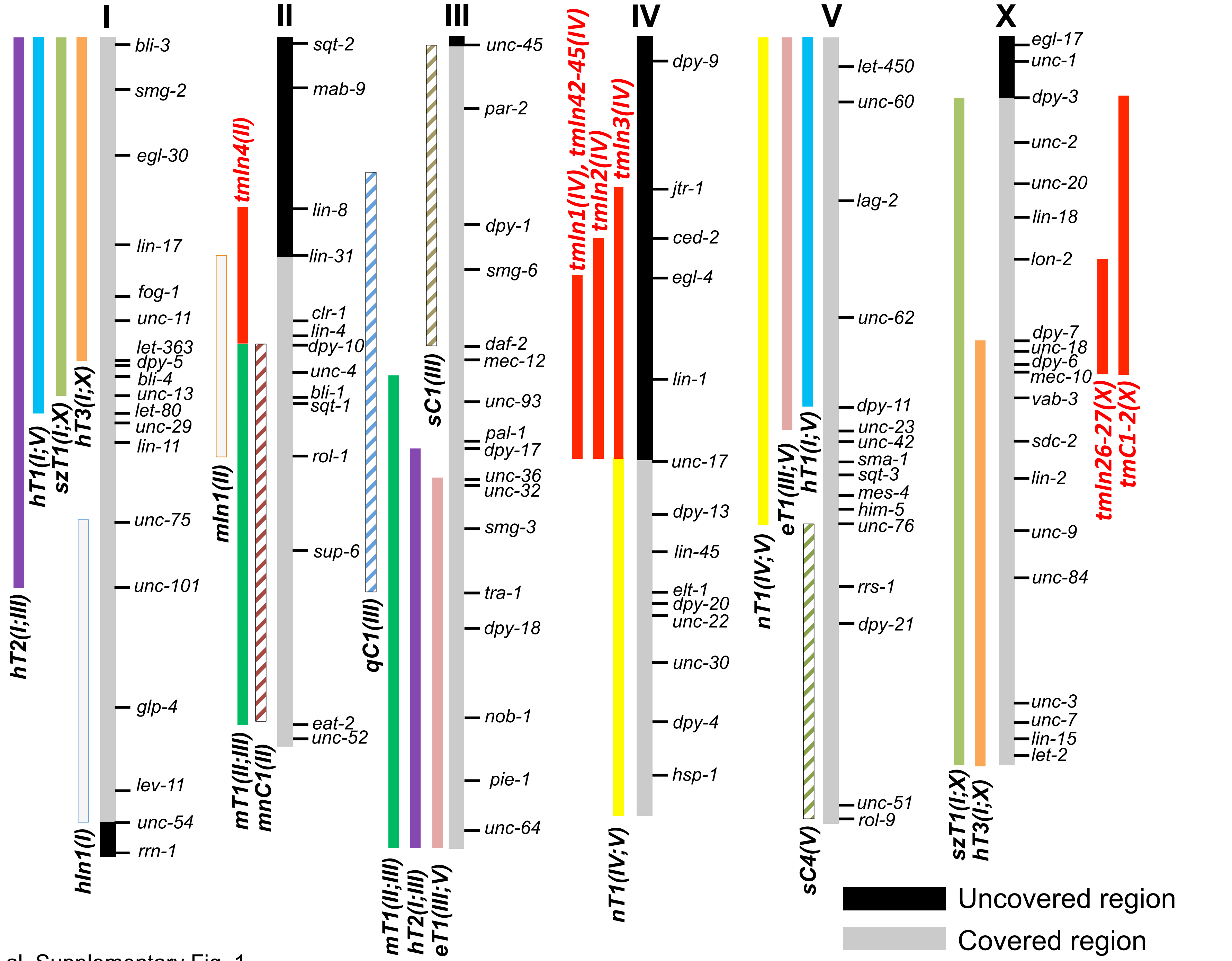

Iwata et al. Supplementary Fig. 1

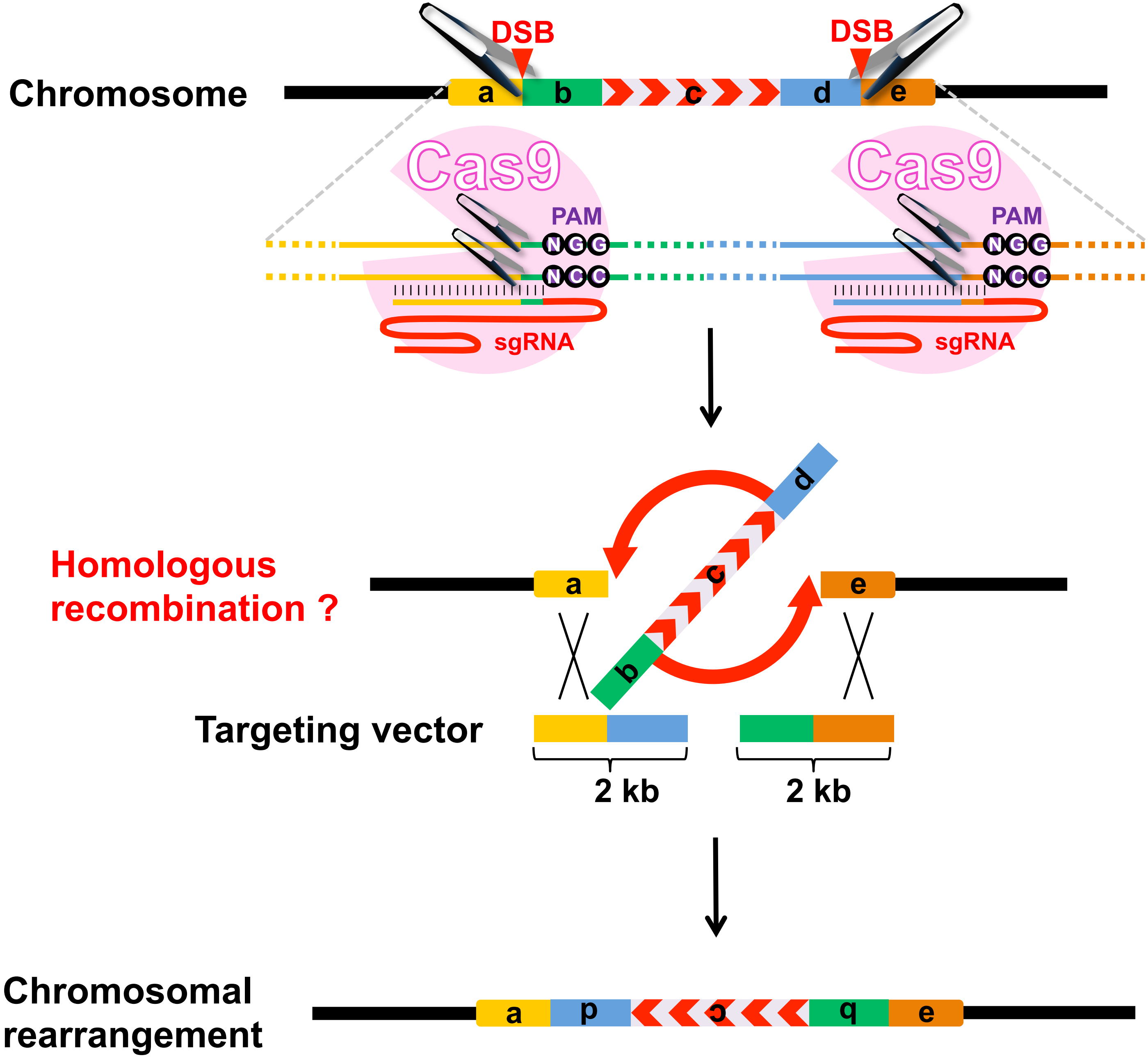

Iwata et al. Supplementary Fig. 2

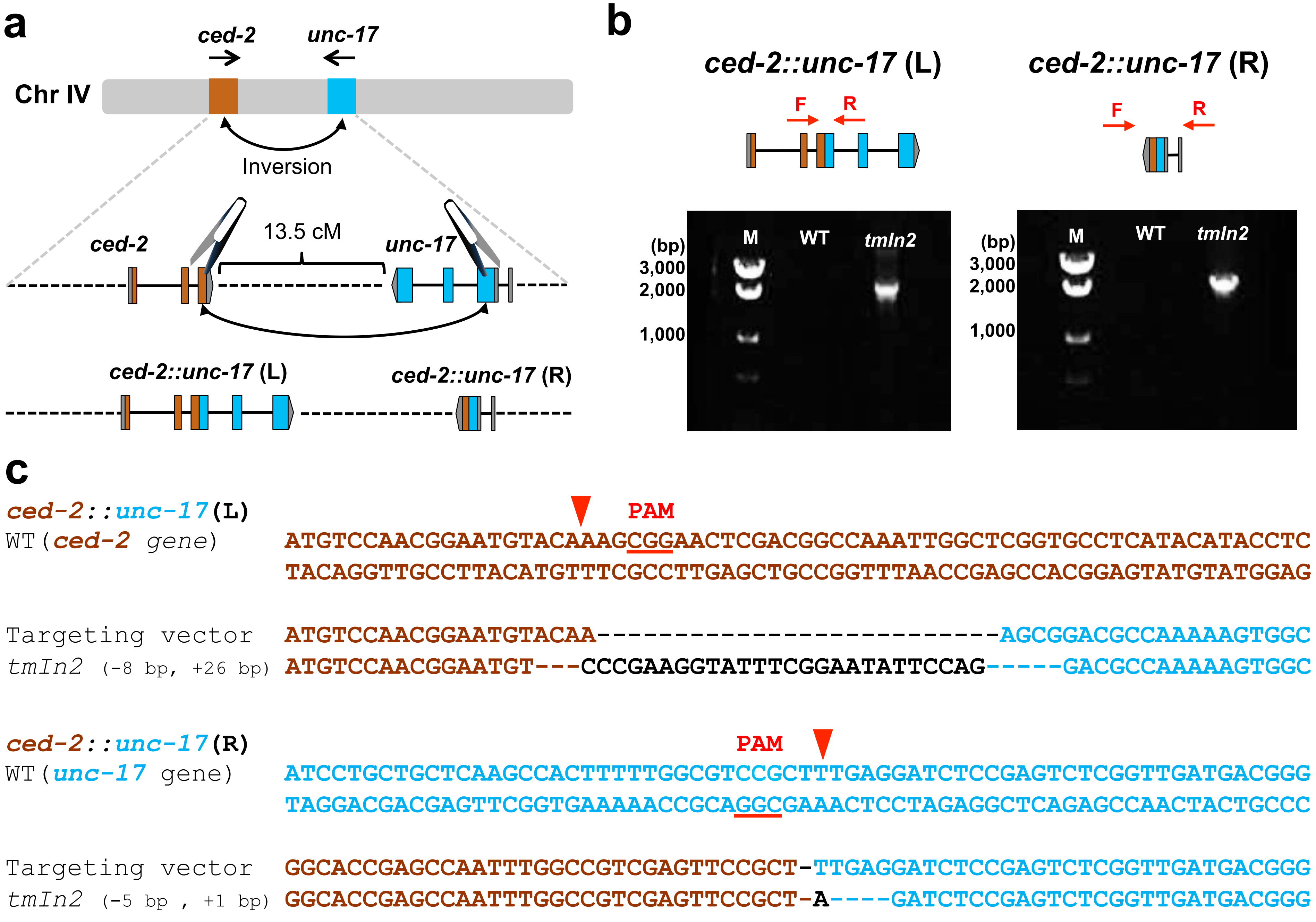

Iwata et al. Supplementary Fig. 3

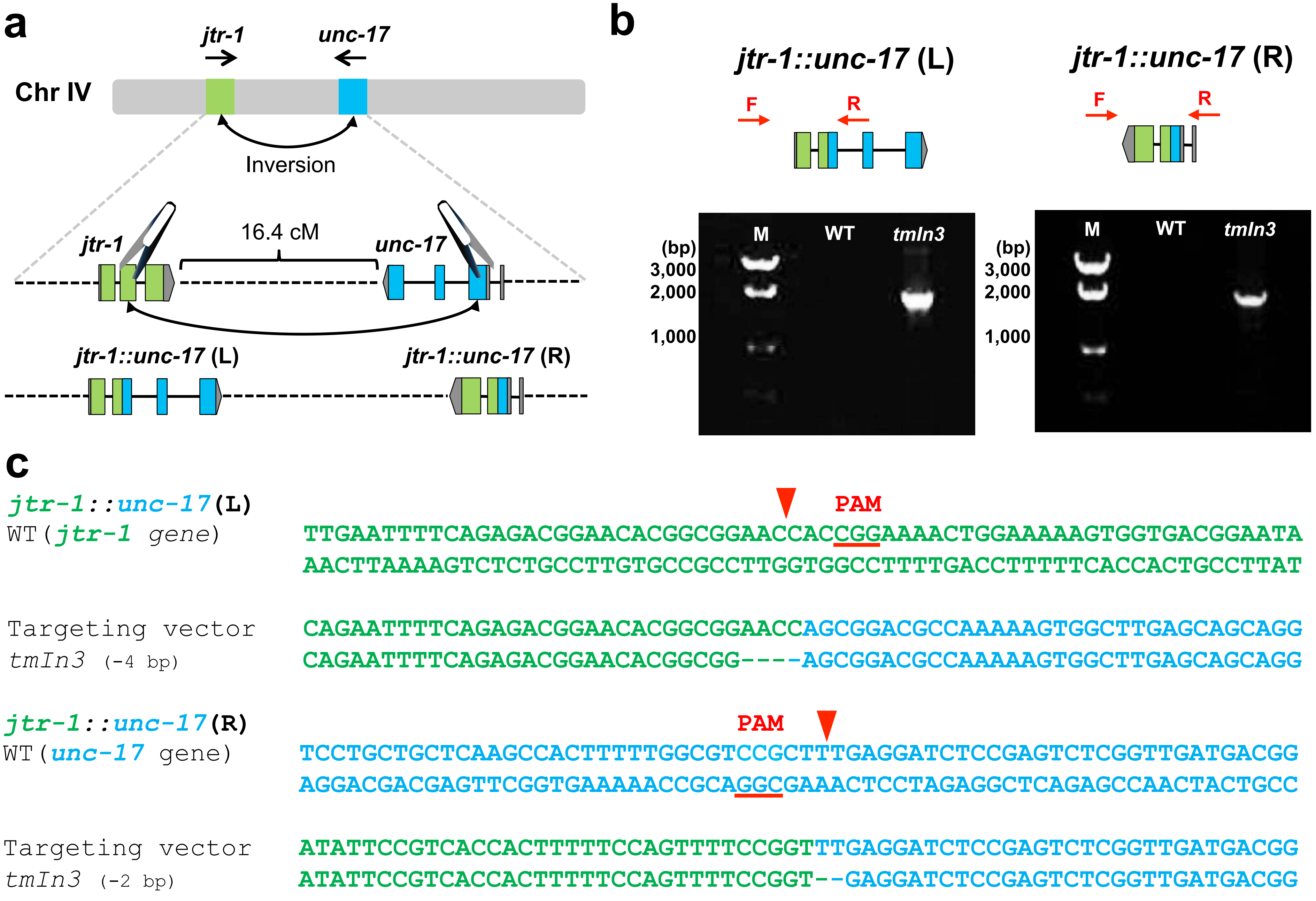

Iwata et al. Supplementary Fig. 4

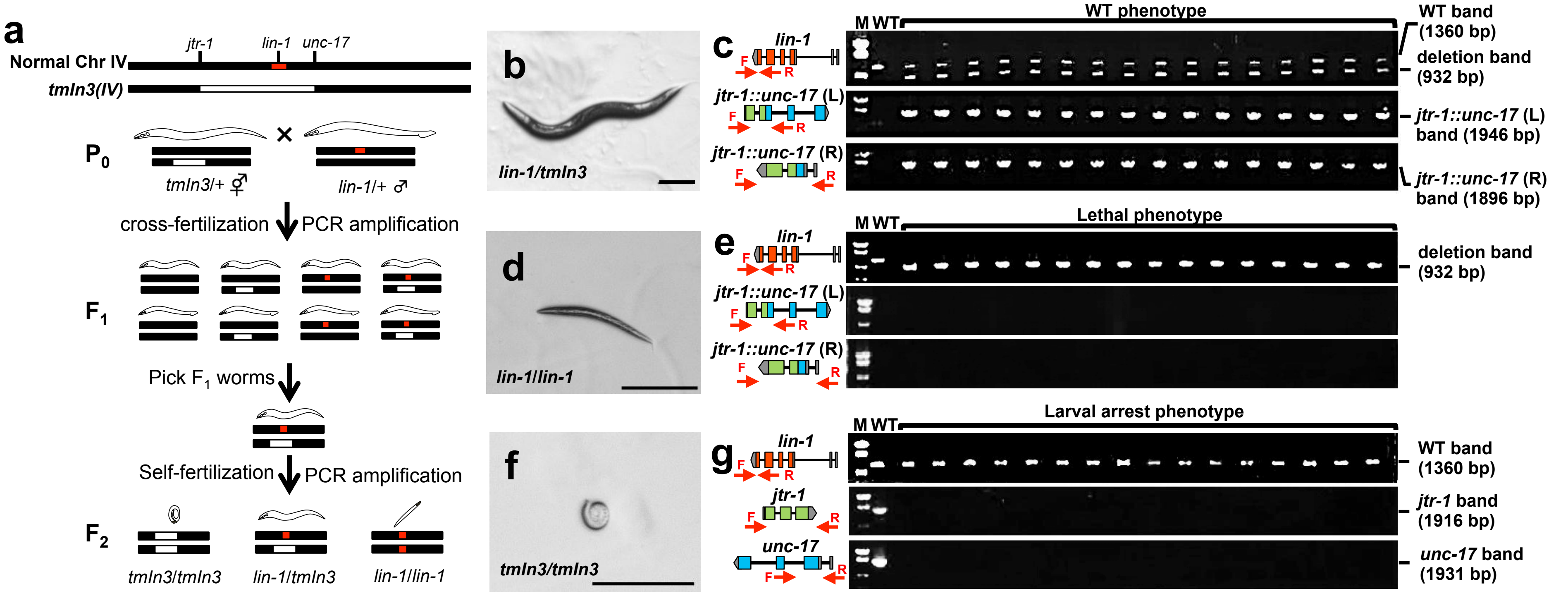

Iwata et al. Supplementary Fig. 5

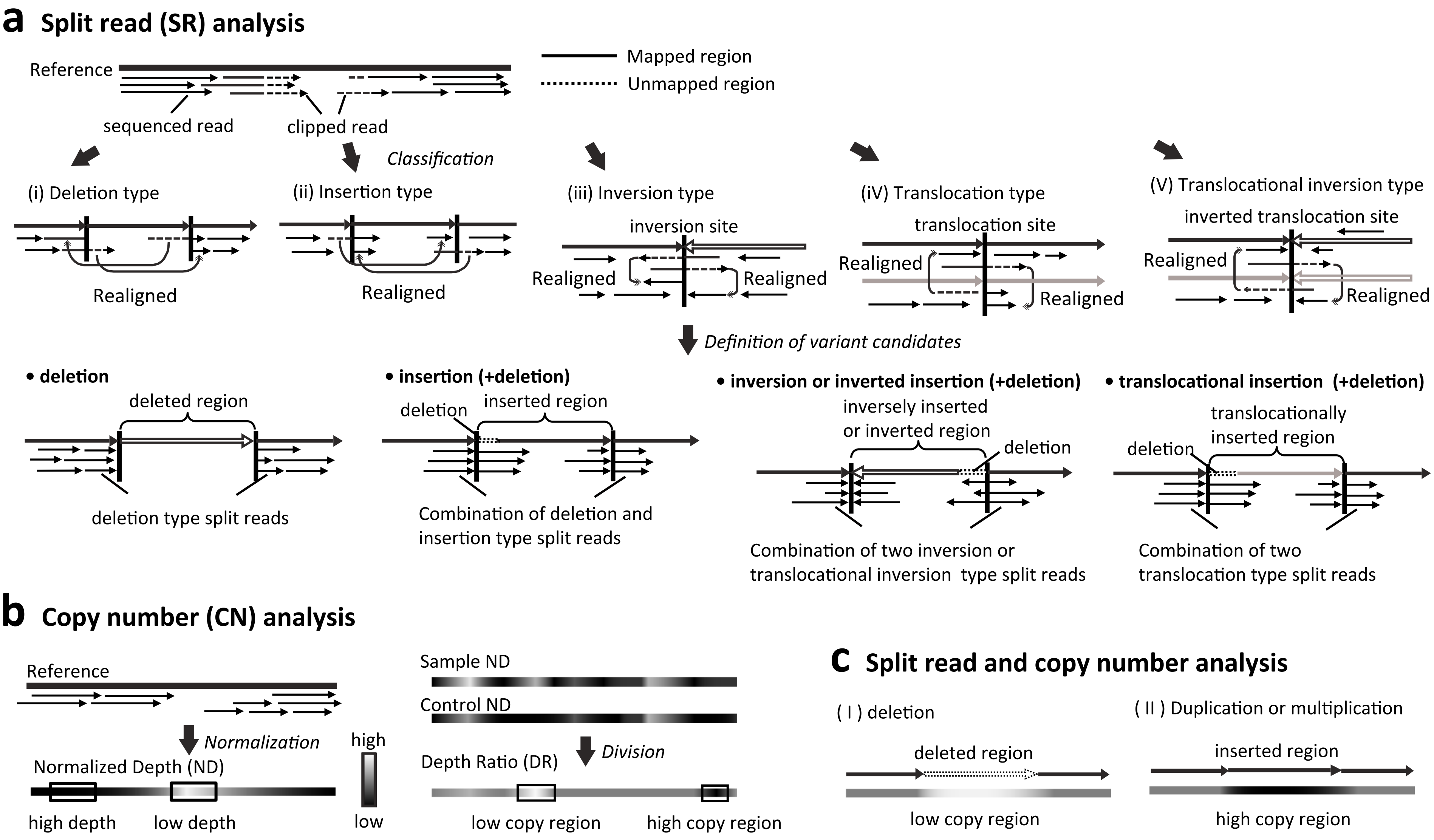

Iwata et al. Supplementary Fig. 6

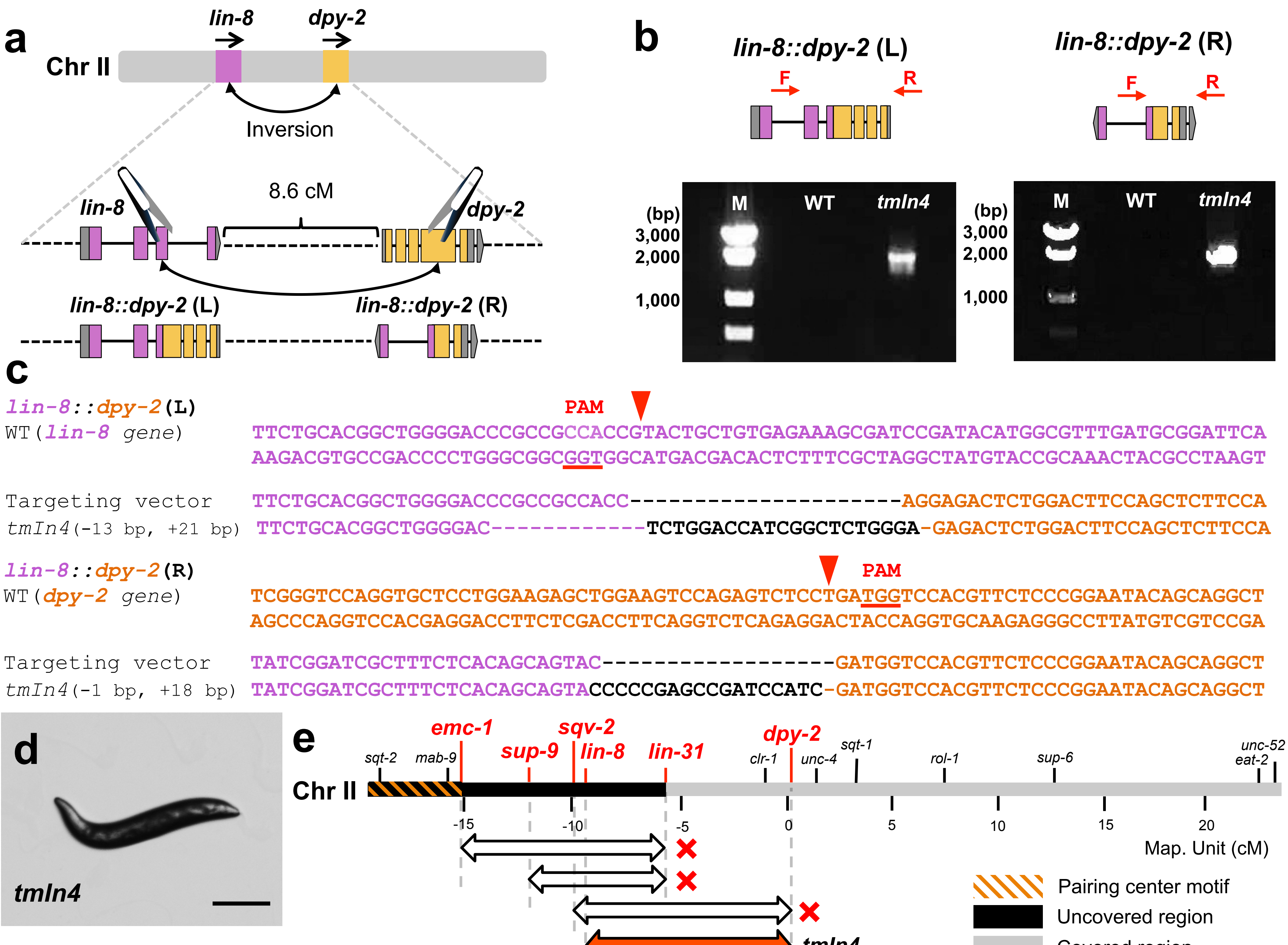

Iwata et al. Supplementary Fig. 7

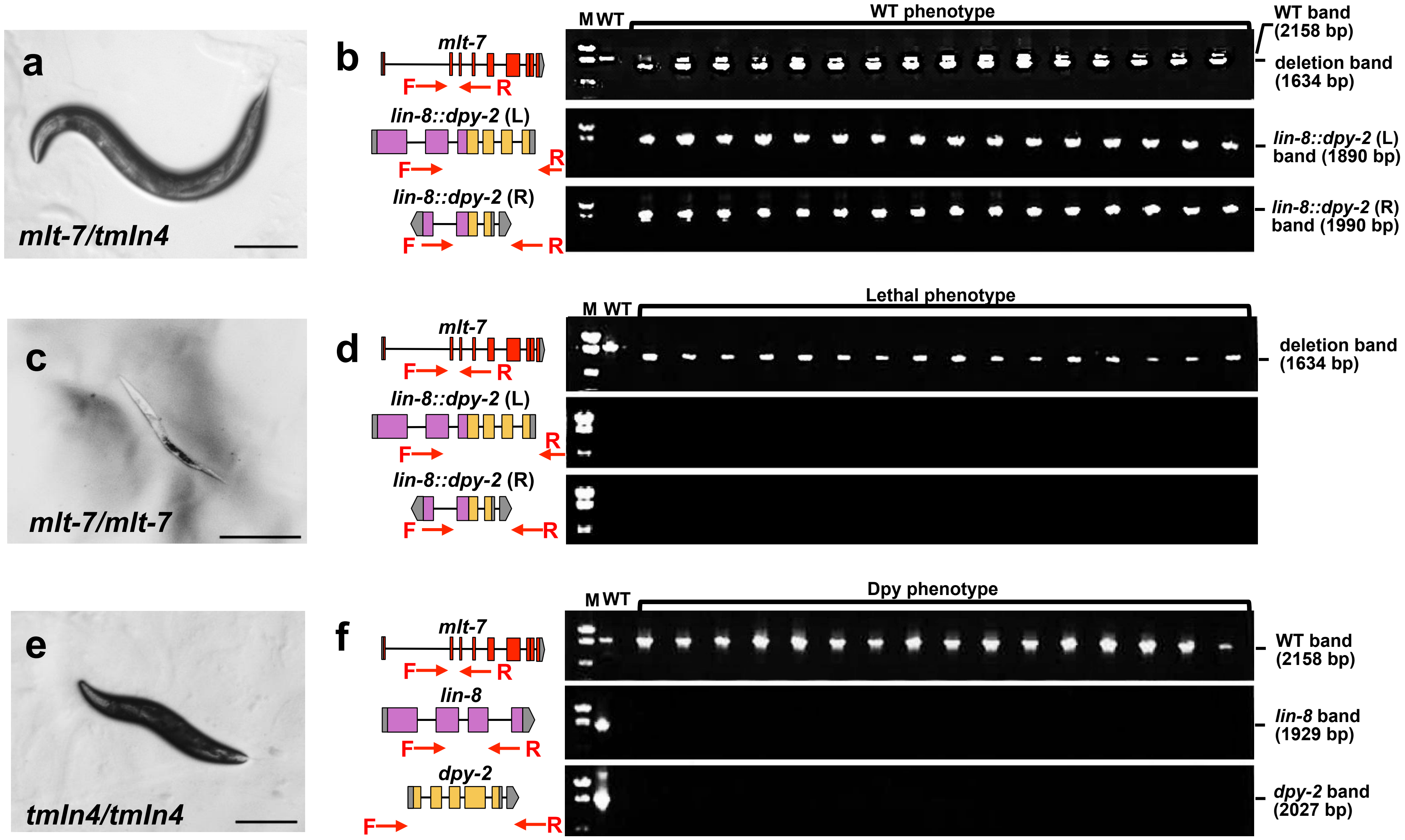

Iwata et al. Supplementary Fig. 8

a

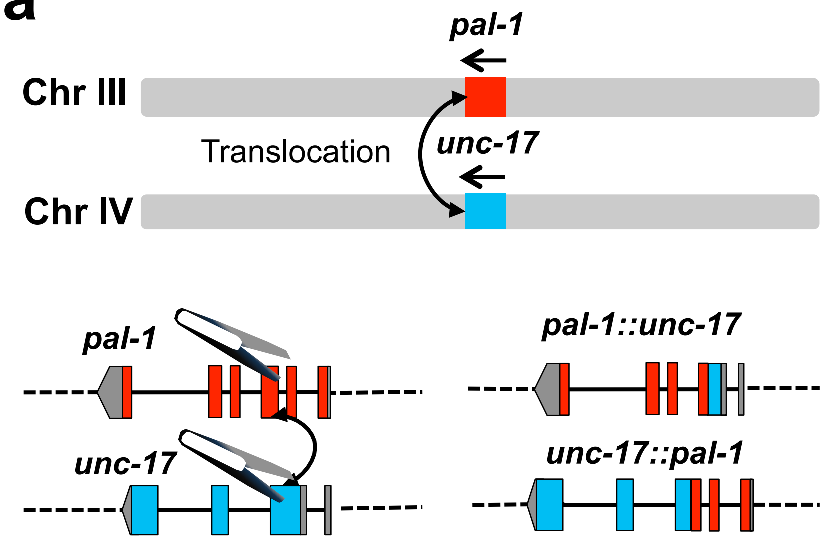

b

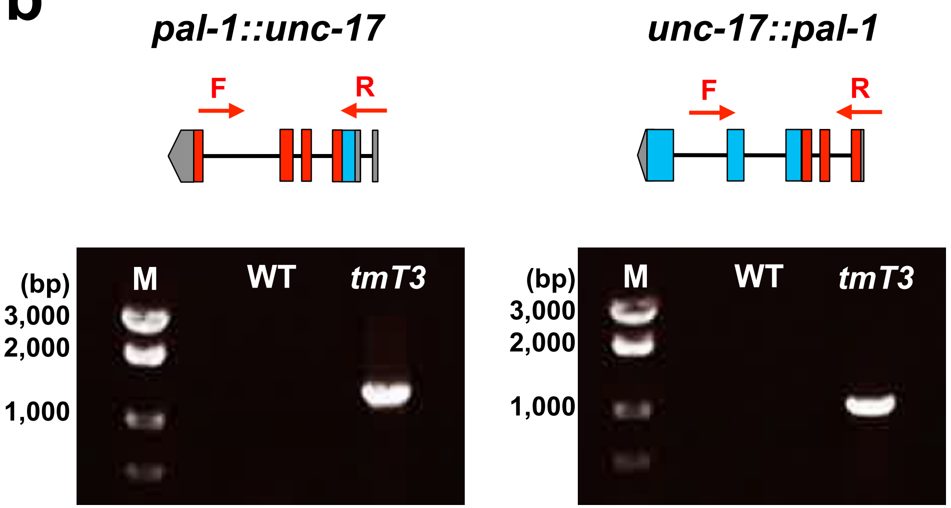

c

pal-1::unc-17

PAM

WT (*pal-1* gene) TGTCATCATATCACTCGGTGATGGATACATTCCACTATCACTACTGCTACTTCCATTCGAAAGCGG

ACAGTAGTATAGTGAGCCACTACCTATGTAAGGTGATAGTGATGACGATGAAGGTAAGCTTTCGCC

Targeting vector TGTCATCATATCACTCGGTGATGGATACATTCCACTTTGAGGATCTCCGAGTCTCGGTTGATGACG

tmT3 (±0 bp) TGTCATCATATCACTCGGTGATGGATACATTCCACTTTGAGGATCTCCGAGTCTCGGTTGATGACG

unc-17::pal-1

PAM

WT (*unc-17* gene) CTGCTCAAGCCACTTTTTGGCGTCCGCTTTGAGGATCTCCGAGTCTCGGTTGATGACGGGCACGT

GACGAGTTCGGTGAAAAACCGCAGGCGAAACTCCTAGAGGCTCAGAGCCAACTACTGCCCGTGCA

Targeting vector CGTGCTCAAGCCACTTTTTGGCGTCCGCTATCACTACTGCTAC ≈ CATTGGATAGTGAGGCCACG

tmT3 (−754 bp) CGTGCTCAAGCCACTTTTTGGCGTCCG---(−751 bp)CATTGGATAGTGAGGCCACG

d

Balancer name	Cas9 targets	Background genotype	P_0 worms	F_1 worms	Phenotype in F_2	F_1 PCR	F_2 PCR	Ratio (%)
tmT3	*pal-1(III) unc-17(IV)*	*lig-4* (*tm750*)	39	197	48	6	1	0.51

Iwata et al. Supplementary Fig. 9

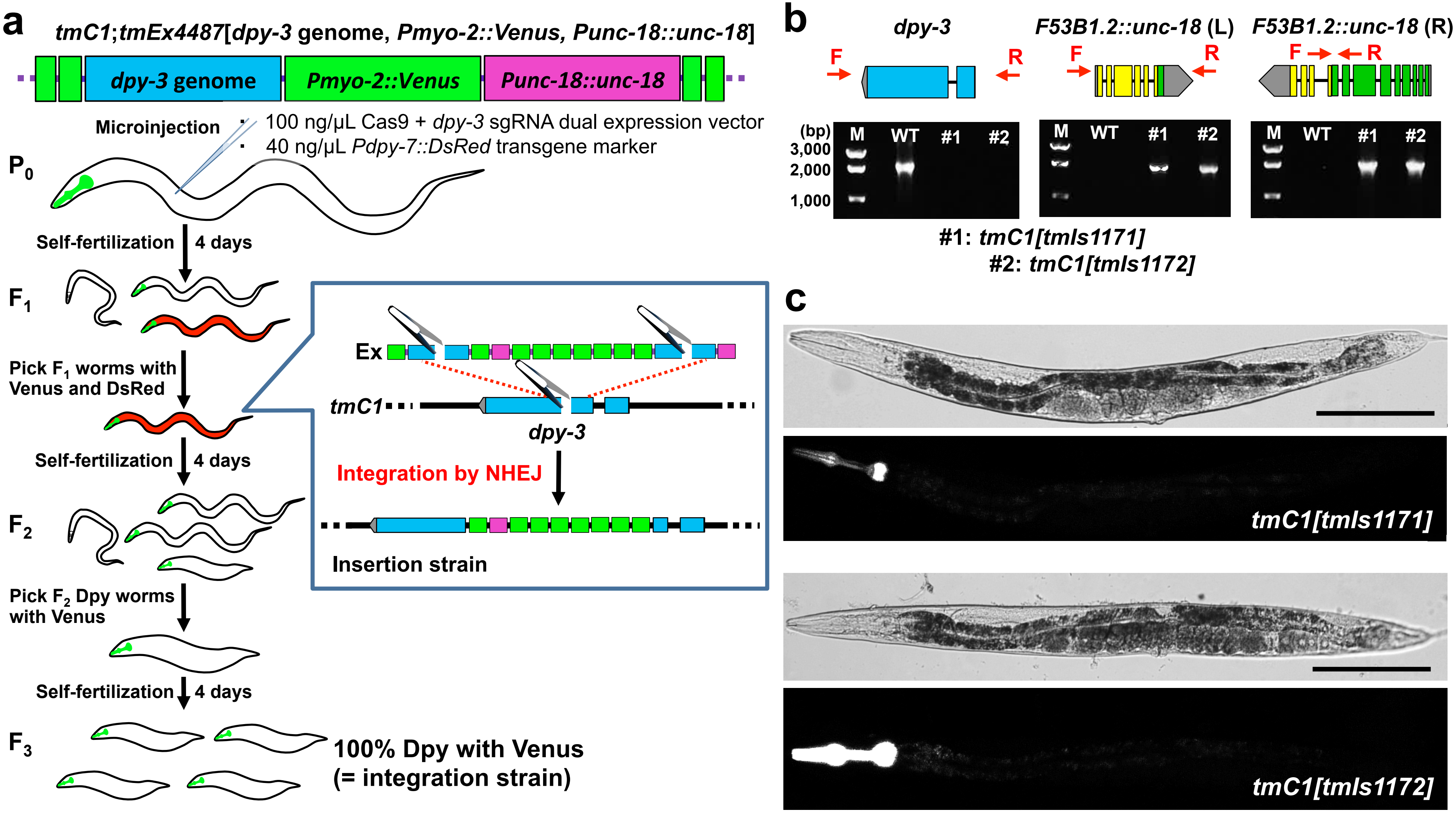

d

Parent strain	Cas9 target	P_0	F_1 (Venus(+) & DsRed(+))	Producing Dpy	Producing Is	Rate (%)
tmC1;tmEx4487 [*dpy-3* genome : *Pmyo-2::Venus* : *Punc-18::unc-18* = 1 : 8 : 1]	*dpy-3*	21	21	5	2	9.5

Iwata et al. Supplementary Fig. 10

egl-4::unc-17(L)

```
                                                            PAM
WT(egl-4 gene)              CCTGTGAAGATTGTCGACGACTTCCGAGAGGAGTTTGCACAGGTTACACTGAAAAATGTGAAGAGGCTAGCGACACTTGGAGTTGGAGGATTTGGA
                            GGACACTTCTAACAGCTGCTGAAGGCTCTCCTCAAACGTGTCCAATGTGACTTTTTACACTTCTCCGATCGCTGTGAACCTCAACCTCCTAAACCT

Predicted sequence          CCTGTGAAGATTGTCGACGACTTCC-------------------------------------------AAGCGGACGCCAAAAAGTGGCTTGAGCA

tmIn42(WT      -4 bp, +12 bp) CCTGTGAAGATTGTCGACGACT---GTCGAATCAACC--------------------------------AGCGGACGCCAAAAAGTGGCTTGAGCA
tmIn43(WT:     -6 bp)        CCTGTGAAGATTGTCGACGA--------------------------------------------------AGCGGACGCCAAAAAGTGGCTTGAGCA
tmIn44(WT:     -2 bp, +43 bp) CCTGTGAAGATTGTCGACGACTTC-TGTCGACTGTCGACATCTTGTCTGACGACTTCTGTCGACTGGG-AGCGGACGCCAAAAAGTGGCTTGAGCA
tmIn45(lig-4:  -2 bp, +33 bp) CCTGTGAAGATTGTCGACGACTTCCGATGGTCCAAACTCGAAAAATCCAAACTGTTTG------------GCGGACGCCAAAAAGTGGCTTGAGCA
```

egl-4::unc-17(R)

```
                                                                                   PAM
WT(unc-17 gene)             GAGACTATCACCAGGACGCATTTCTTCTGATTATCCTGCTGCTCAAGCCACTTTTTGGCGTCCGCTTTGAGGATCTCCGAGTCTCGGTTGATGACG
                            CTCTGATAGTGGTCCTGCGTAAAGAAGACTAATAGGACGACGAGTTCGGTGAAAAACCGCAGGCGAAACTCCTAGAGGCTCAGAGCCAACTACTGC

Predicted sequence          TTTTTCAGTGTAACCTGTGCAAACTCCTCTC------------------------------------TGAGGATCTCCGAGTCTCGGTTGATGACG

tmIn42(WT:     -1 bp, +14 bp) TTTTTCAGTGTAACCTGTGCAAACTCCTCTCGATTCGACGATGTC-----------------------GAGGATCTCCGAGTCTCGGTTGATGACG
tmIn43(WT:     -6 bp,  +1 bp) TTTTTCAGTGTAACCTGTGCAAACTC-----A------------------------------------GAGGATCTCCGAGTCTCGGTTGATGACG
tmIn44(WT:     -1 bp, +36 bp) TTTTTCAGTGTAACCTGTGCAAACTCCTCTCGTCGAGCGTGAAGATTGTGAAGATTGTCGACTTGAC-GAGGATCTCCGAGTCTCGGTTGATGACG
tmIn45(lig-4: -32 bp)        TTTTTCAGTGTAACC--------------------------------------------------------------------TCGGTTGATGACG
```

Iwata et al. Supplementary Fig. 11
